# A threshold level of NFATc1 activity facilitates thymocyte differentiation and opposes notch-driven leukaemia development

**DOI:** 10.1038/ncomms11841

**Published:** 2016-06-17

**Authors:** Stefan Klein-Hessling, Ronald Rudolf, Khalid Muhammad, Klaus-Peter Knobeloch, Muhammad Ahmad Maqbool, Pierre Cauchy, Jean-Christophe Andrau, Andris Avots, Claudio Talora, Volker Ellenrieder, Isabella Screpanti, Edgar Serfling, Amiya Kumar Patra

**Affiliations:** 1 Department of Molecular Pathology, Institute for Pathology, University of Wuerzburg, Josef-Schneider Strasse 2, 97080 Wuerzburg, Germany; 2 Department of Pathology, Institute of Neuropathology, University Clinic Freiburg, Breisacher Strasse 64, 79106 Freiburg, Germany; 3 Institute de Génétique Moléculaire de Montpellier (IGMM), UMR5535 CNRS, 1919 Route de Mende, 34293 Montpellier Cedex 5, France; 4 Institute of Biomedical Research, College of Medical and Dental Sciences, University of Birmingham, Birmingham B15 2TT, UK; 5 Laboratory of Molecular Pathology, Department of Molecular Medicine, Sapienza University of Rome, Viale Regina Elena 291, 00161 Rome, Italy; 6 Department of Gastroenterology II, University of Goettingen, Robert Koch Strasse 40, 37075 Goettingen, Germany

## Abstract

NFATc1 plays a critical role in double-negative thymocyte survival and differentiation. However, the signals that regulate *Nfatc1* expression are incompletely characterized. Here we show a developmental stage-specific differential expression pattern of *Nfatc1* driven by the distal (P1) or proximal (P2) promoters in thymocytes. Whereas, preTCR-negative thymocytes exhibit only P2 promoter-derived *Nfatc1β* expression, preTCR-positive thymocytes express both *Nfatc1β* and P1 promoter-derived *Nfatc1α* transcripts. Inducing NFATc1α activity from P1 promoter in preTCR-negative thymocytes, in addition to the NFATc1β from P2 promoter impairs thymocyte development resulting in severe T-cell lymphopenia. In addition, we show that NFATc1 activity suppresses the B-lineage potential of immature thymocytes, and consolidates their differentiation to T cells. Further, in the pTCR-positive DN3 cells, a threshold level of NFATc1 activity is vital in facilitating T-cell differentiation and to prevent Notch3-induced T-acute lymphoblastic leukaemia. Altogether, our results show NFATc1 activity is crucial in determining the T-cell fate of thymocytes.

## Abstract (web summary)

NFATc1 orchestrates thymocyte development. Here the authors show that NFATc1 expression is regulated by distinct promoters during thymocyte differentiation, and by conditional deletion of individual promoters in mice they define their specific roles in the control of T-cell development by NFATc1.

## Graphical abstract

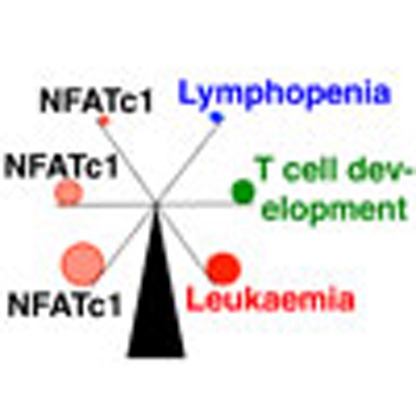

Differentiation of CD4^−^CD8^−^ double-negative (DN) cells to the CD4^+^ or CD8^+^ single-positive (SP) T cells in the thymus is regulated by a complex network of signalling pathways involving multiple transcription factors at various stages of development. On the basis of their differentiation status, DN thymocytes consist of four distinct populations, CD44^+^CD25^−^DN1, CD44^+^CD25^+^DN2, CD44^−^CD25^+^DN3 and CD44^−^CD25^−^DN4 (ref. 1). DN3 thymocytes upon rearrangement of their T-cell receptor β (*Tcrb*) locus express a functional TCRβ chain, which, in combination with the preTα (pTα) chain forms the preTCR (pTCR). PreTCR signalling is essential for the differentiation of DN3 cells to DN4 and later stages. We have shown recently that the transcription factor NFATc1 plays a critical role during early stages of thymocyte development^2^. A haematopoietic lineage cell-specific ablation of NFATc1 activity blocked thymocyte development at DN1 stage, the earliest stage of thymic T-cell development^2^. Mice deficient for NFATc2, NFATc3 or both, do not show any apparent defect in thymocyte development^3^,^4^, suggesting a critical role played by NFATc1 during thymic T-cell development.

Ligation of TCR with cognate peptide-MHC complex *in vivo*, or stimulation of T cells with anti-CD3 plus anti-CD28 Abs *in vitro* increases intracellular Ca^2+^ levels, which in turn activate the serine/threonine phosphatase calcineurin. Active calcineurin dephosphorylates multiple serine/threonine residues in NFAT proteins and facilitates their nuclear translocation. We have previously elucidated a novel NFAT activation pathway in pTCR-negative thymocytes, which plays an indispensable role in their survival and differentiation^2^. In contrast to the calcineurin-mediated dephosphorylation pathway, in pTCR-negative thymocytes IL-7-JAK3 signals activated NFATc1 via phosphorylation of tyrosine^371^ in the regulatory domain^2^. Both, the calcineurin-mediated ‘conventional' and IL-7-JAK3-mediated ‘alternative' pathways though explained the post-translational mechanisms of NFAT activation, the transcriptional regulation of *Nfatc1* itself is poorly understood. A previous study showed *Nfatc1* expression in T cells is autoregulated by NFATc1 (ref. 5). In this study, we have delineated the signalling pathways that regulate *Nfatc1* expression with distinct promoter usage at pTCR-negative and -positive thymocytes. Further, we provide evidence in support of a critical role of NFATc1 in suppressing lineage plasticity of immature thymocytes towards non-T lineages, and the essentiality of a threshold level of NFATc1 activity at the pTCR-positive DN3 stage in facilitating the T-cell fate of thymocytes by preventing the development of T-Acute Lymphoblastic Leukaemia (T-ALL).

## Results

### Differential usage of *Nfatc1* promoters in thymocytes

**Figure 1:**
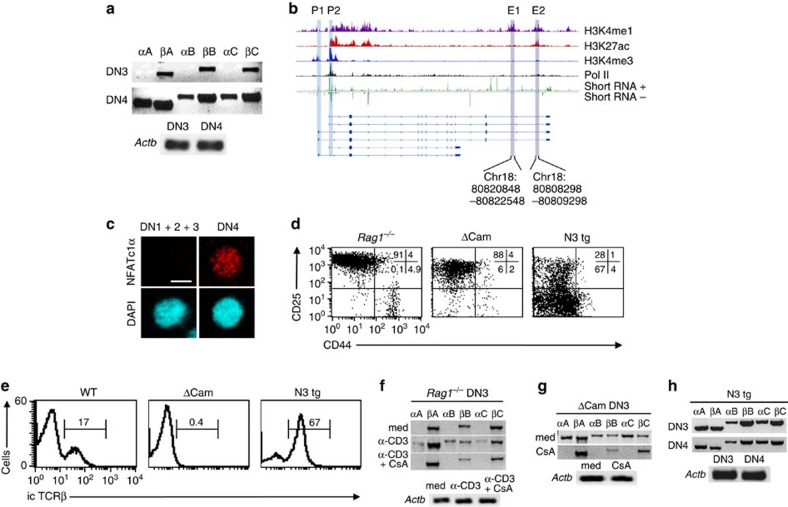
Differential induction of *Nfatc1* P1 and P2 promoters in pTCR-negative and -positive thymocytes. (**a**) RT–PCR analysis of *Nfatc1* isoforms expression in WT DN3 and DN4 cells. (**b**) ChIP-Seq and small RNA-Seq analysis of *Nfatc1* gene for histone methylation, acetylation, RNA Polymerase II binding and bidirectional transcriptional start site RNA at the P1 and P2 promoter region in *Rag2*^−/−^ DN3 cells. Blue rectangles highlight promoters, whereas pink rectangles show putative enhancer hallmarks based on the presence of epigenetic/transcriptional features. Chromosomal coordinates represented below are the ones corresponding to DNase I hypersensitive sites described in Fig. 6a that are both inside the highlighted areas. (**c**) Immunofluorescence analysis of NFATc1α distribution in WT DN3 and DN4 cells. Nuclear staining was revealed by DAPI. Scale bar, 10 μm. (**d**) Flow cytometry profiles of DN1 to DN4 cells distribution in indicated mice. (**e**) Intracellular TCRβ staining in WT, ΔCam and N3 tg DN3 cells. (**f**) RT–PCR analysis reveals the expression of six *Nfatc1* isoforms in DN3 cells from *Rag1*^−/−^ mice either freshly sorted, or treated with anti-CD3 Abs in absence or presence of CsA for 12 h. (**g**) Transcript levels of six *Nfatc1* isoforms in freshly isolated or 12 h CsA-treated DN3 cells from ΔCam mice. (**h**) Pattern of *Nfatc1* isoforms expression in freshly sorted DN3 and DN4 cells from N3 tg mice. Numbers within each dot plot or histogram represent per cent respective population. Data are representative of three independent experiments.

In T cells, two distinct promoters, a distal (P1) and a proximal (P2), initiate *Nfatc1* expression. Due to alternative splicing and usage of two different poly-adenylation (pA) sites, six *Nfatc1* isoforms; three from P1 promoter (*Nfatc1*αA, αB and αC) and three from P2 promoter (*Nfatc1*βA, βB and βC), respectively, are synthesized (Supplementary Fig. 1a)^6^,^7^. To distinguish whether any particular NFATc1 isoform is prevalent in pTCR-negative and -positive thymocytes we analysed wild-type (WT) DN1–DN4 cells for *Nfatc1* expression. Interestingly, we observed an exclusive P2 promoter activity in the pTCR-negative DN3 cells, whereas pTCR-positive DN4 cells exhibited both P2 as well as P1 promoter activity (Fig. 1a; Supplementary Fig. 1b). Exclusive P2 activity in pTCR-negative thymocytes was supported by an active chromatin configuration, as indicated by histone modifications and a concomitant recruitment of RNA polymerase II (Pol II) at the *Nfatc1* P2 promoter in *Rag2*^−/−^ DN3 cells, whereas only little H3K4me3 was detectable at the P1 promoter (Fig. 1b). Analysis of small RNA-seq data^8^, to score for bidirectional paused transcripts enriched at promoters further confirmed significant enrichment of transcriptional start site RNAs at P2 promoter over P1 (Fig. 1b). Using an additional set of primers to detect total α- or β-specific transcripts derived from the P1 or P2 promoters respectively, again we observed an exclusive P2 activity in DN3, and both P1 and P2 activity in DN4 cells (Supplementary Fig. 1c). Further, in agreement with our observation regarding induction of P1 promoter activity at the pTCR-positive stages, we detected NFATc1α proteins in DN4 cells but not in DN3 cells (Fig. 1c; Supplementary Fig. 1d). Keeping in view the findings of a previous report^5^, that in naive T cells, constitutive basal level TCR signalling maintains the P2 promoter activity, and TCR-antigen ligation induced signals are responsible for the P1 promoter activity, it was quite intriguing how thymocytes lacking even the pTCR exhibit such a robust *Nfatc1* expression.

To further substantiate our observation regarding selective promoter usage, and the role of pTCR signalling in inducing P1 activity, we investigated three different mouse models, where either there was no pTCR (*Rag1*^−/−^), pTCR was absent but a downstream signalling molecule calcineurin was constitutively active (Calcineurin transgenic; ΔCam)^9^ or there was enhanced pTCR signalling (Notch3 transgenic; N3 tg)^10^. In both *Rag1*^−/−^ and ΔCam mice, T-cell development is blocked at the DN3 stage due to lack of pTCR signals^9^. In contrast, N3 tg mice showed an enhanced DN3 to DN4 transition due to strong pTCR signals (Fig. 1d,e). Confirming our ChIP-Seq and RNA-Seq observations, analysis of *Nfatc1* expression in *Rag1*^−/−^ DN3 cells showed the presence of only *Nfatc1*β isoforms (P2 activity), similar to that in WT DN3 cells (Fig. 1f). Stimulating *Rag1*^−/−^ DN3 cells with anti-CD3 Abs to mimic pTCR signalling, we readily detected *Nfatc1*α transcripts in addition to the *Nfatc1*β transcripts implying that pTCR signals can induce P1 promoter activity (Fig. 1f). Further, P1 promoter activity was autoregulated by NFATc1, as P1 activity disappeared without affecting P2 activity when anti-CD3 antibodies stimulated *Rag1*^−/−^ DN3 cells were treated with cyclosporine A (CsA; Fig. 1f). In contrast to the WT and *Rag1*^−/−^ mice, ΔCam DN3 cells showed a robust P1 activity in addition to the P2 promoter activity (Fig. 1g). The strong P1 activity in ΔCam DN3 cells was due to the autoregulatory loop maintained by constitutive calcineurin activity, as CsA treatment specifically extinguished *Nfatc1* P1 activity without affecting the P2 activity (Fig. 1g). Further, we observed a similar pattern of strong P1 and P2 promoter activity as that in ΔCam mice, in N3 tg DN3 cells (Fig. 1h). Thus, it was evident that *Nfatc1* is expressed from distinct promoters in pTCR-negative and -positive thymocytes, and pTCR signalling is necessary for the induction of *Nfatc1* P1 promoter activity.

### NFATc1 activity is vital for DN thymocyte differentiation

**Figure 2:**
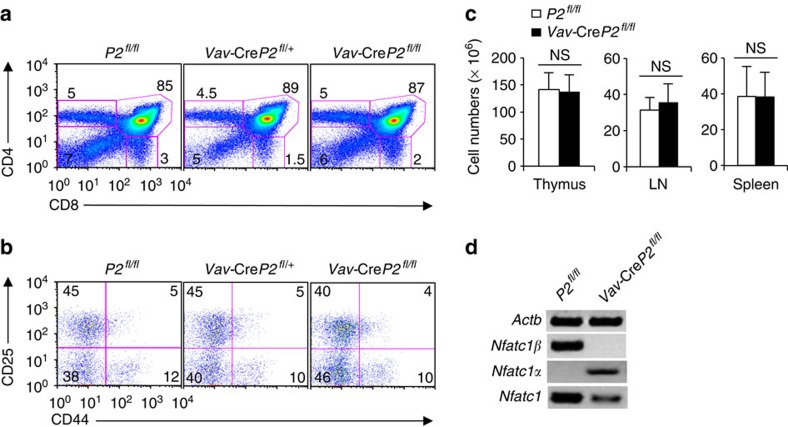
*Nfatc1* P1 promoter activity compensates for the loss of P2 activity in T-cell development. (**a**) Distribution of thymocyte subsets based on CD4 and CD8 expression in *P2*^fl/fl^, *Vav-*Cre*P2*^fl/+^ and *Vav-*Cre*P2*^fl/fl^ mice. (**b**) DN1–DN4 cells distribution within DN thymocytes of *Vav-*Cre*P2*^fl/fl^ mice compared with that in littermate control mice based on CD44 and CD25 expression. (**c**) Cellularity in thymus, LNs and Spleen from *P2*^fl/fl^ (*n*=10) and *Vav*-Cre*P2*^fl/fl^ (*n*=11) mice. (**d**) RT–PCR analysis for *Nfatc1*, *Nfatc1*α and *Nfatc1*β expression in pTCR-negative (DN1+2+3) thymocytes from *P2*^fl/fl^ and *Vav*-Cre*P2*^fl/fl^ mice (*n*=8 per group). Data are representative of three independent experiments and are shown as mean±s.d., NS, not significant, unpaired *t*-test.

Due to exclusive P2 activity in pTCR-negative thymocytes, we investigated whether NFATc1β is solely critical for the differentiation of early DN thymocytes. To clarify this, we have generated a mutant mouse with floxed *Nfatc1* P2 promoter element (*P2*^fl/fl^) to abolish NFATc1β activity in a tissue-specific manner (Supplementary Fig. 2a–d). We bred *P2*^fl/fl^ mice with mice expressing cre-recombinase under *Vav* promoter (*Vav*-Cre)^11^ to abolish *Nfatc1* P2 activity during thymocyte development. Surprisingly, analysis of *Vav*-Cre*P2*^fl/fl^ mice showed completely normal T-cell development, which was indistinguishable from that of littermate controls (Fig. 2a–c; Supplementary Fig. 2e). The normal T-cell development in *Vav*-Cre*P2*^fl/fl^ mice was quite surprising, as loss of NFATc1 activity was expected to block thymocyte development at the DN1 stage^2^. PCR with reverse transcription (RT–PCR) analysis on pTCR-negative thymocytes showed a total lack of *Nfatc1* P2-derived transcripts indicating abolition of *Nfatc1* P2 promoter activity (Fig. 2d). However, NFATc1 activity was not lost rather in absence of P2 activity we observed a robust P1 promoter activity in the *Vav*-Cre*P2*^fl/fl^ pTCR-negative thymocytes (Fig. 2d).

We have previously shown that Bcl-2 is a target of NFATc1 in pTCR-negative thymocytes^2^. However, analysis in *Vav*-Cre*P2*^fl/fl^ DN3 cells showed similar levels Bcl-2 proteins as that in littermate controls (Supplementary Fig. 2f), suggesting that most likely NFATc1α and -β isoforms regulate a similar set of target genes that are critical for early thymocyte survival and differentiation. Thus, in the *Vav*-Cre*P2*^fl/fl^ mice, *Nfatc1* P1 promoter activity functionally compensated for the loss of P2 activity, underlining the indispensability of NFATc1 activity in thymocyte differentiation.

### NFATc1 activity is essential for T-cell development

**Figure 3:**
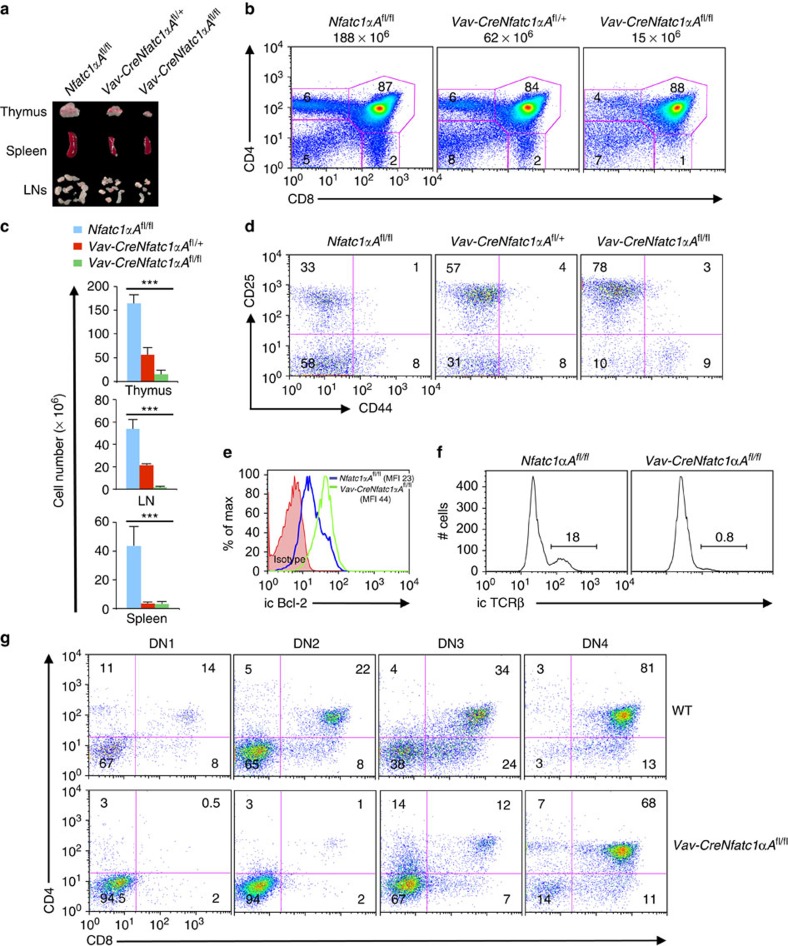
*Nfatc1* P1 activity in addition to P2 activity in pTCR-negative cells impairs thymocyte differentiation. (**a**) Photograph showing the dosage-dependent effect of *Nfatc1* P1 activity on the size of the thymus, LNs and spleen in *Vav*-Cre*Nfatc1*αA^fl/+^ and *Vav*-Cre*Nfatc1*αA^fl/fl^ mice compared with *Nfatc1*αA^fl/fl^ control mice. (**b**) Distribution of thymocyte subsets based on CD4 and CD8 expression in indicated mice. Number above each plot represents total thymic cellularity. (**c**) Cellularity in thymus, LNs and spleen from *Nfatc1*αA^fl/fl^, *Vav*-Cre*Nfatc1*αA^fl/+^ and *Vav*-Cre*Nfatc1*αA^fl/fl^ mice (*n*=10 per group). (**d**) Distribution of DN1–DN4 cells based on CD44 and CD25 expression among DN thymocytes from indicated mice. (**e**) Intracellular Bcl-2 expression in *Vav*-Cre*Nfatc1*αA^fl/fl^ DN3 cells compared with that in *Nfatc1*αA^fl/fl^ control mice. MFI, mean fluorescence index. (**f**) Intracellular TCRβ expression in DN3 cells from indicated mice. Number inside each plot represents per cent TCRβ-positive cells. (**g**) Impaired differentiation of *Vav*-Cre*Nfatc1*αA^fl/fl^ DN1–DN4 cells to DP stage on OP9-DL1 monolayer compared with WT cells *in vitro*. Numbers inside each plot represent per cent respective population. Data represent one of three independent experiments (*n*=4 per group), and are shown as mean±s.d., ^***^P<0.0001, one-way analysis of variance.

To investigate the physiological significance of the distinct pattern of NFATc1 promoter activity in pTCR-negative and -positive thymocytes, we explored what impact NFATc1α will have on thymocyte development if it is co-expressed with NFATc1β in pTCR-negative thymocytes. To address this issue, we used mice in which a constitutively active version of *Nfatc1*α*A* was knocked-in into *Rosa-26* locus flanked by a floxed stop cassette (*R26-caNfatc1*αA-Stop^fl/fl^: designated hereafter as *Nfatc1*αA^fl/fl^)^12^. To activate *Nfatc1*α*A* expression in early thymocytes we bred *Nfatc1*αA^fl/fl^ mice with *Vav*-Cre mice. Surprisingly, analysis of *Vav*-Cre*Nfatc1*αA^fl/fl^ mice showed severely impaired thymocyte development as evident from a dose-dependent reduction in the size of the thymus, spleen and lymph nodes (Fig. 3a). Accordingly, the cellularity in these organs was drastically reduced in *Vav*-Cre*Nfatc1*αA^fl/fl^ mice leading to T-cell lymphopenia (Fig. 3b,c). Analysis of DN cells from *Vav*-cre*Nfatc1*αA^fl/fl^ mice to understand the reason behind the low thymic cellularity revealed a dose-dependent block in the transition of DN3 cells to the DN4 stage (Fig. 3d; Supplementary Fig. 3a). Confirming our earlier report regarding NFATc1-mediated regulation of Bcl-2 expression in developing DN thymocytes, enforced NFATc1α expression enhanced Bcl-2 levels in Vav-Cre*Nfatc1*αA^fl/fl^ DN3 cells compared with WT cells (Fig. 3e). This observation further suggests that the reduced thymic cellularity in the *Vav*-cre*Nfatc1*αA^fl/fl^ mice was not because of an increase in cell death of the DN thymocytes. Similar to the ΔCam mice^9^, enforced NFATc1αΑ expression in DN3 cells resulted in the lack of rearranged TCRβ expression (Fig. 3f), leading to the loss of pTCR signalling, although the expression of CD3ɛ a component of the pTCR complex remained unaffected (Supplementary Fig. 3b). In addition, NFATc1αA activity in pTCR-negative cells strongly retarded their rate of differentiation to the double-positive (DP) and later stages. In *in vitro* co-culture assays on OP9-DL1 (bone marrow stromal cells expressing Notch ligand delta-like 1) monolayer, *Vav*-Cre*Nfatc1*αA^fl/fl^ DN1–DN4 cells showed inefficient differentiation to DP stage compared with the cells from WT mice (Fig. 3g). These observations suggest that the combined activity of NFATc1α and NFATc1β, or rather an increase in total NFATc1 activity, before pTCR-positive stages is detrimental for T-cell development.

To rule out the possibility that the negative impact on thymocyte development in the *Vav*-Cre*Nfatc1*αA^fl/fl^ mice was due to an excess in NFATc1 activity derived from both the endogenous as well as the transgene, we generated a mouse model where only NFATc1αΑ was expressed without any contribution from the endogenous *Nfatc1* gene. We bred *Vav*-Cre*Nfatc1*αA^fl/fl^ mice with *Nfatc1*^fl/fl^ mice to eliminate all NFATc1 activity derived from the endogenous *Nfatc1* gene. Analysis of *Vav-CreNfatc1αΑ*^fl/fl^*Nfatc1*^fl/fl^ mice revealed that even in the absence of any endogenous NFATc1, NFATc1α activity derived from the knocked-in gene only resulted in a similar block in thymocyte differentiation at the DN3 stage compared with littermate controls (Supplementary Fig. 3c,d), as observed in the *Vav-CreNfatc1αΑ*^fl/fl^ mice. This could still be due to an above threshold level of NFATc1 activity derived from the transgene. Thus, from our analysis it is apparent that a certain threshold level of NFATc1 activity is essential for T-cell development.

### NFATc1 suppresses lineage plasticity of immature thymocytes

**Figure 4:**
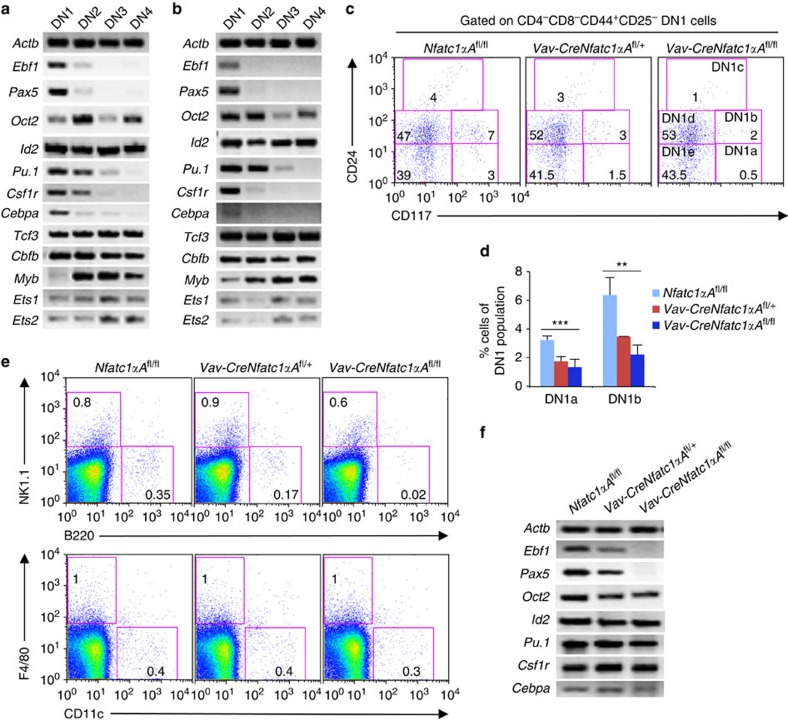
NFATc1 activity suppresses B-lineage potential of immature thymocytes. (**a**) RT–PCR analysis of B, NK, macrophage and DC lineage specifying genes expression in DN1–DN4 cells from WT mice. (**b**) Gene expression analysis on cells as mentioned in **a** from Vav-Cre*Nfatc1P2*^fl/fl^ mice. (**c**) Analysis of DN1a-e cells distribution based on CD117 and CD24 expression among DN1 cells from *Nfatc1*αA^fl/fl^, Vav-Cre *Nfatc1*αA^fl/+^ and Vav-Cre *Nfatc1*αA^fl/fl^ mice. (**d**) Distribution of DN1a and DN1b cells within DN1 population in Vav-Cre*Nfatc1*αA^fl/fl^ (*n*=6) and Vav-Cre*Nfatc1*αA^fl/+^ (*n*=6) mice compared to control *Nfatc1*αA^fl/fl^ (*n*=4) mice. (**e**) Flow cytometry profiles reveal the distribution of B, NK, macrophage and DC populations in the thymus of indicated mice. (**f**) RT–PCR analysis of B, NK, macrophage and DC lineage specifying genes expression in DN1 cells from Vav-Cre *Nfatc1*αA^fl/fl^ mice compared with Vav-Cre *Nfatc1*αA^fl/+^ and *Nfatc1*αA^fl/fl^ control mice. Numbers inside each plot represent per cent respective population. Data are representative of three independent experiments and are shown as mean±s.d., ***P=0.0007 and **P=0.0013, one-way analysis of variance.

T-cell development in the thymus follows a sequential process of T-lineage specification, and commitment. While DN1 cells retain the potential to differentiate into B cells, natural killer (NK) cells, dendritic cells (DCs) and macrophages, the lineage plasticity gets restricted to NK and DC lineages in DN2 cells, and finally is completely lost at the DN3 stage^13^. Accordingly, only in WT DN1 cells we observed *Ebf1* and *Pax5* expression necessary for B-cell development, whereas, expression of *Id2* necessary for NK cell, or *Spf1* (PU.1), *Csf1r* and *Cebpa*, necessary for myeloid-lineage development were maintained in DN2 cells (Fig. 4a). We assumed that NFATc1β activity most likely play a role in suppressing the lineage plasticity during DN1 to DN3 differentiation and thereby consolidate T-lineage commitment. To investigate this possibility, we analysed lineage-specific gene expression in DN1–DN4 cells from *Vav*-Cre*P2*^fl/fl^ mice. We observed a similar suppression of lineage plasticity towards non-T lineages in the absence of NFATc1β activity (Fig. 4b), as in the WT mice (Fig. 4a). As in the *Vav*-Cre*P2*^fl/fl^ mice P1 promoter-driven NFATc1α activity compensated for the loss of NFATc1β during T-cell development, we concluded that a threshold level of NFATc1 activity irrespective of any particular isoform might be crucial in suppressing lineage plasticity and thereby facilitate T-lineage commitment.

DN1 cells are a heterogeneous population, and based on CD24 (heat-stable antigen) and CD117 (c-Kit) expression they are characterized to have five distinct populations termed as DN1a, DN1b, DN1c, DN1d and DN1e (ref. 13). DN1a and DN1b cells maintain highest lineage plasticity, whereas, DN1d and DN1e cells are more specified to take the T-cell lineage. We presumed that the combined activity of NFATc1α, and NFATc1β is essential for irreversible T-lineage commitment at the pTCR-positive stage, and NFATc1α if expressed along with NFATc1β early at the pTCR-negative stages will suppress the lineage plasticity more effectively. Interestingly, analysis of Vav-Cre*Nfatc1*αA^fl/fl^ mice showed a strong reduction in DN1a and DN1b populations compared with littermate WT mice (Fig. 4c,d). Accordingly, we observed a dose-dependent specific loss of B cells in the thymus from Vav-Cre*Nfatc1*αA^fl/fl^ mice compared to WT mice whereas, NK, DC or macrophage lineages were not affected (Fig. 4e). Corroborating this, *Ebf1* and *Pax5* expression were suppressed in Vav-Cre*Nfatc1*αA^fl/fl^ DN1 cells, whereas gene expression necessary for NK and myeloid lineages was unaffected (Fig. 4f). However, the suppression of B-lineage potential of the Vav-Cre*Nfatc1*αA^fl/fl^ DN1 cells was neither due to enhanced cell death nor due to any toxic effect of NFATc1 activity on the precursor cells. In a B-lineage permissible environment, FACS sorted DN1 thymocytes from *Vav-CreNfatc1αΑ*^fl/fl^ mice developed comparable proportion of B220^+^ B cells as that in case of WT DN1 cells, when co-cultured on OP9 bone marrow stromal cell layer (Supplementary Fig. 3e). This observation ruled out that there was any inherent developmental restriction towards B-lineage differentiation in the Vav-Cre*Nfatc1*αA^fl/fl^ DN1 cells.

### Integrin signalling induces *Nfatc1* P2 promoter activity

**Figure 5:**
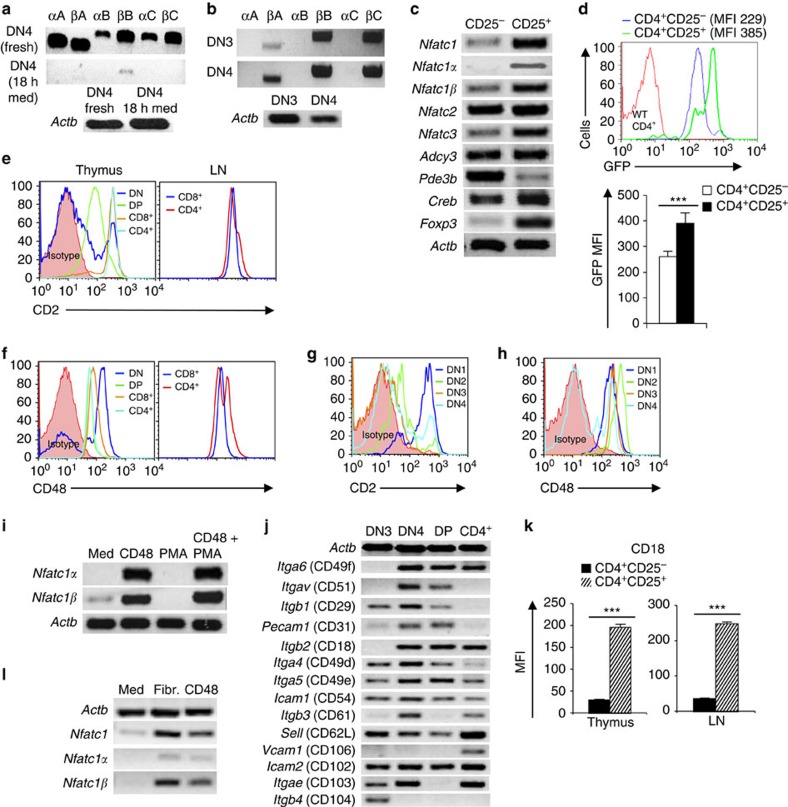
Integrin-cyclic-AMP signalling regulates *Nfatc1* P2 activity. (**a**) RT–PCR analysis of *Nfatc1* isoforms expression in WT DN4 cells cultured in medium only for 18 h compared with that in freshly isolated DN4 cells. (**b**) RT–PCR analysis of *Nfatc1* isoforms expression in 8-CPT-cAMP treated WT DN3 and DN4 cells. (**c**) Analysis of *Nfatc1*, *Nfatc1*α, *Nfatc1*β and other genes in WT CD4^+^CD25^+^ T_reg_ cells compared with CD4^+^CD25^-^ T_eff_ cells. (**d**) Analysis of GFP expression levels in thymic T_eff_ and T_reg_ cells from *Nfatc1-eGfp-Bac* tg mice, and the quantification of their mean GFP fluorescence intensities. (**e**) CD2 expression levels in WT DN, DP, CD4^+^ and CD8^+^ thymocytes and CD4^+^ or CD8^+^ T cells from LNs. (**f**) CD48 expression on cells as mentioned in **e**. (**g**,**h**) Flow cytometry analysis of WT DN1–DN4 cells for CD2 (**g**) and CD48 (**h**) expression, respectively. (**i**) RT–PCR analysis of *Nfatc1*α and *Nfatc1*β expression in WT DN3 cells stimulated with CD48 Abs (5 μg) or PMA (100 ng) alone or in combination of both for 18 h. (**j**) Gene expression analysis for various integrins in WT DN3, DN4, CD4^+^CD8^+^ DP and CD4^+^ SP cells from thymus as revealed by RT–PCR. (**k**) CD18 expression levels (MFI) in T_reg_ and T_eff_ cells from thymus and LNs of WT mice, (*n*=4). (**l**) Analysis of *Nfatc1* expression pattern in fibronectin (Fibr.; 1 μg) or CD48Abs (0.5 μg) stimulated peripheral CD4^+^ T cells. Data are representative of three independent experiments and are shown as mean±s.d., *P<0.0001, paired *t*-test.

The exclusive P2 activity in the WT pTCR-negative thymocytes led us to ask how the P2 promoter is regulated in these cells. Surprisingly, when we cultured DN4 thymocytes *in vitro*, not only all P1-directed transcripts disappeared but we also observed the absence of all P2-derived transcripts as well (Fig. 5a). However, annexin V analysis revealed that majority of cells in this culture condition were alive, ruling out cell death being the reason for the loss of *Nfatc1* expression (Supplementary Fig. 4a). We have previously reported the responsiveness of *Nfatc1* promoter activity to Forskolin (FSK) treatment *in vitro*^5^. To investigate whether cyclic-AMP (cAMP) signalling is responsible for *Nfatc1* P2 activity, we treated WT DN3 and DN4 cells with a cAMP analogue 8-CPT-cAMP. Interestingly, 8-CPT-cAMP treatment specifically induced the expression of *Nfatc1*β, both in DN3 and DN4 cells (Fig. 5b). To prove that P2 activity is cAMP signalling-dependent, we explored for a physiological context where the intracellular cAMP level is high. Several recent studies have reported increased cAMP levels in CD4^+^CD25^+^Foxp3^+^ regulatory T (T_reg_) cells^14^,^15^,^16^. The effect of cAMP in inducing *Nfatc1* P2 promoter activity was confirmed as T_reg_ cells exhibited stronger P2 promoter activity compared with CD4^+^CD25^-^Foxp3^-^ effector T (T_eff_) cells (Fig. 5c). This observation was corroborated with higher level of green fluorescent protein (GFP) expression reflecting higher NFATc1 levels in T_reg_ cells against T_eff_ cells from *Nfatc1*-*eGfp*-*Bac* tg reporter mice^17^ (Fig. 5d).

Next, we investigated which signalling pathway is involved in generating intracellular cAMP that drives *Nfatc1* expression in thymocytes and in T cells. Cell–cell interaction is mediated by various integrins, and thymocytes, as well as T cells express a number of integrins. CD2 (LFA-2) expressed on thymocytes and T cells has been shown to activate T cells^18^,^19^. Murine CD2 binds to its ligand CD48 expressed on T cells as well as on the interacting cells and thereby can transduce signals both in *cis* and in *trans*^20^,^21^,^22^,^23^. In addition, CD2 signalling in T cells have been reported to induce cAMP^24^,^25^, and thereby the activation of cAMP response element binding (CREB) proteins^26^. Mice lacking cAMP-CREB signalling show impaired thymocyte proliferation and altered fetal T-cell development^27^,^28^. To check whether CD2-CD48 interactions regulate *Nfatc1* expression in DN thymocytes, we analysed CD2 and CD48 expression on various thymocyte subsets. We observed only a fraction of DN thymocytes expressed CD2 on their surface, which was increased in DP and SP thymocytes as well as in peripheral T cells (Fig. 5e). However, CD48 expression was highest on DN cells and lowest in DP and CD4^+^ SP cells (Fig. 5f). Among DN thymocytes, DN1 cells expressed the highest level of CD2, whereas CD48 expression was high on all pTCR-negative DN populations (Fig. 5g,h). To prove whether CD2-CD48 signals could induce *Nfatc1* expression, we stimulated WT DN3 cells with CD48 antibodies and analysed for the generation of P1- and P2-directed transcripts. CD48 stimulation at a higher concentration induced the synthesis of both P1- and P2-directed *Nfatc1* transcripts in DN3 cells, while stimulation with PMA, reported to substitute CD2 co-stimulation^29^, did not generate any *Nfatc1* RNA (Fig. 5i). However, CD48 at low concentration only induced *Nfatc1* P2 activity in the pTCR-negative thymocytes unraveling the effect of signal strength on the inducibility of P1 and P2 promoters (Supplementary Fig. 4b).

Although CD2-CD48 signalling induced *Nfatc1* expression *in vitro*, these are unlikely to be the only integrins responsible for *Nfatc1* expression *in vivo*. This was supported by the reported normal T-cell development in *Cd2*^−/−^ mice^30^,^31^,^32^, as well as in mice injected with anti-CD2 antibodies^33^, and also in *Cd48*^−/−^ mice^34^, indicating the involvement of additional integrins in regulating *Nfatc1* expression. Accordingly, analysis of thymocytes and T cells showed a differential expression pattern for various integrins (Supplementary Fig. 4c; Fig. 5j). CD4^+^CD25^+^ T_reg_ cells both in the thymus and in LNs expressed much higher levels of various integrins compared to CD4^+^CD25^−^ T_eff_ cells (Fig. 5k; Supplementary Fig. 4d), concurring with our observation regarding higher *Nfatc1* expression in T_reg_ cells over T_eff_ cells (Fig. 5c). Among the CD4^+^CD25^+^ population, CD25^hi^ cells expressed highest level of various integrins, the most prominent being CD18, CD48, CD49d and CD62L (Supplementary Fig. 5a,b). This integrin expression pattern directly correlated to that of *Nfatc1* expression in CD25^hi^ cells as revealed from higher GFP levels in the *Nfatc1*-*eGfp*-*Bac* transgenic reporter mice (Supplementary Fig. 5c). To further consolidate our observation, we stimulated WT CD4^+^ T cells with fibronectin, the ligand for CD18. Interestingly, fibronectin stimulation induced robust *Nfatc1* expression, which was mainly derived from the P2 promoter (Fig. 5l). In addition, similar to that in DN3 thymocytes (Fig. 5i), CD2-CD48 signalling also induced *Nfatc1* P2 activity in peripheral CD4^+^ T cells (Fig. 5l). Thus, the above observations established integrin-cAMP signalling as a critical component in regulating *Nfatc1* gene expression in thymocytes and in T cells.

### An intronic *cis*-regulatory element controls P1 activity

**Figure 6:**
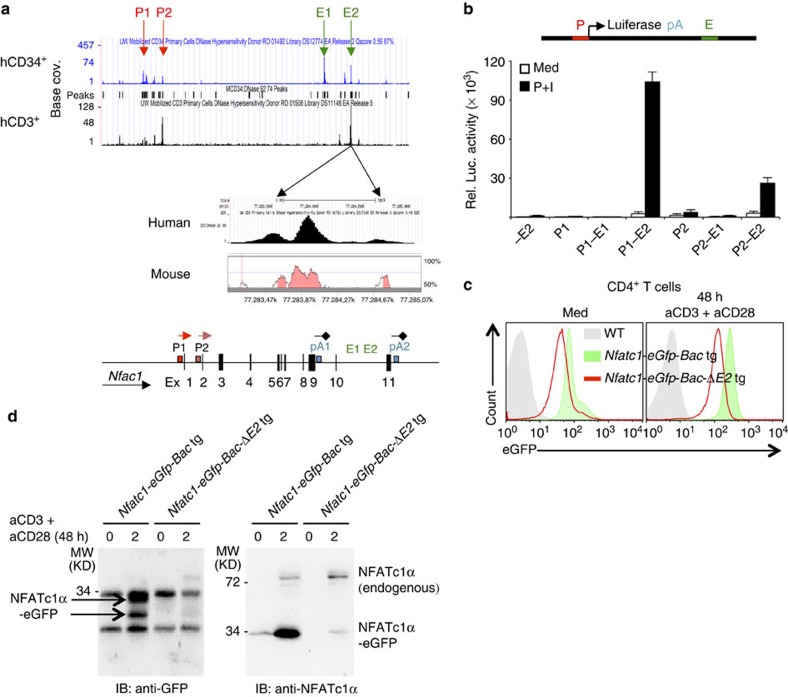
*Nfatc1* P1 activity is induced by a novel enhancer element. (**a**) DNase I hypersensitive sites in the *NFATc1* locus in human hematopoietic stem and CD3^+^ T cells. Their corresponding position in the murine *Nfatc1* gene is also shown. (**b**) Enhancer activity of the E2 element in inducing *Nfatc1* P1 promoter activity in reporter assays. (**c**) NFATc1 expression levels as revealed by GFP expression in freshly isolated, or aCD3+aCD28 Abs stimulated CD4^+^ T cells from *Nfatc1-eGfp-Bac*-*ΔE2* tg mice compared with that from *Nfatc1-eGfp-Bac* tg mice. (**d**) Immunoblot analysis for GFP or NFATc1α expression in unstimulated and 48 h aCD3+aCD28 Abs stimulated CD4^+^ T cells from *Nfatc1-eGfp-Bac*-*ΔE2* tg mice compared with that from *Nfatc1-eGfp-Bac* tg mice.

To address the question why P1 promoter is inactive in the pTCR-negative thymocytes, we analysed DNA methylation status at the P1 promoter in these cells. However, methylation analysis did not reveal any significant difference between WT DN3 and DN4 cells (Supplementary Fig. 6a). To investigate whether epigenetic modifications on pTCR signalling at the DN3 to DN4 transition induce P1 activity we performed ChIP-Seq analysis of *Rag2*^−/−^ DN3 cells stimulated with anti-CD3 Abs to mimic pTCR signals. However, no additional epigenetic changes were detected at the P1 promoter in the anti-CD3 stimulated *Rag2*^−/−^ DN3 cells compared to the unstimulated cells (Supplementary Fig. 6b; Fig. 1b). These observations suggested that P1 promoter induction still requires involvement of additional *cis*-regulatory elements and/or *trans*-acting factors. To identify *cis*-regulatory elements essential for P1 promoter activity, we studied the DNase I hypersensitivity analysis of the human *NFATc1* locus (Roadmap Epigenomics Project, www.roadmapepigenomics.org). Besides the P1 and P2 promoter elements, two very distinct DNase I hypersensitive sites designated as E1 and E2 were evident in the intron between exons 10 and 11 in the CD34^+^ human haematopoietic stem cells (Fig. 6a). Significantly, these elements were tissue-specific as compared with the hematopoietic stem cells, CD3^+^ human T cells showed only the presence of E2 site, which was conserved in murine T cells as well (Fig. 6a).

To investigate the influence of E1 and E2 elements on *Nfatc1* expression, we generated luciferase reporter constructs with the P1 or P2 promoter in combination with the E1 or E2 element (Fig. 6b). On stimulation with PMA+Ionomycin (I), in EL-4 thymoma cells the E2, but not the E1 element induced a strong *Nfatc1* P1 promoter activity compared with a mild increase in P2 promoter activity (Fig. 6b). We also tested two additional DNAse I hypersensitive sites, E3 positioned in the intron between exons 3 and 4, and E4 positioned at the extreme 3′ region of *Nfatc1* locus for their potential regulatory effect on *Nfatc1* promoter activity. However, both these elements were ineffective in inducing *Nfatc1* P1 promoter activity (Supplementary Fig. 6c), suggesting the essentiality of the E2 element in the context of P1 promoter activity.

To extend our characterization of the E2 enhancer, we analysed for the *trans*-acting factors, which might play a role in enhancing the E2-mediated P1 promoter activity. We detected binding motifs for three prominent lymphoid-specific transcription factors; NFATc1, GATA3 and PU.1 in the E2 element (Supplementary Fig. 6d). To investigate whether any of these factors positively modulates the E2 enhancer activity, we mutated the binding sites for individual factors and checked their enhancer potential for *Nfatc1* P1 promoter activity. In reporter assays, only the NFATc1 mutant showed loss of enhancer activity, whereas GATA3 or PU.1 mutants exerted no influence on *Nfatc1* P1 promoter activity (Supplementary Fig. 6d). This was in line with the previous reports from our laboratory that NFATc1 autoregulates the P1 promoter activity^5^, however, the involvement of the E2 element was hitherto unknown.

Further, to study the effect of E2 element on *Nfatc1* promoter *in vivo*, we generated *Nfatc1*-*eGfp*-*Bac*-*ΔE2* tg reporter mice by deleting 1Kb DNA from intron 10 harbouring the E2 element (Supplementary Fig. 6e)^17^. Loss of E2 enhancer activity in *Nfatc1*-*eGfp*-*Bac*-*ΔE2* tg reporter mice, showed a clear reduction in *Nfatc1* expression in CD4^+^ T cells as evident from reduced GFP expression both in unstimulated as well as in stimulated cells compared with that in the *Nfatc1*-*eGfp*-*Bac* tg reporter mice (Fig. 6c). This reduction in NFATc1 levels was due to a specific loss of P1-derived NFATc1α, as we observed a strong decrease in NFATc1α proteins in immunoblot analysis with antibodies against GFP as well as NFATc1α, whereas loss of E2 enhancer activity did not influence NFATc1β protein levels (Fig. 6d; Supplementary Fig. 7). Thus, we have identified a novel *cis*-regulatory element E2, acting as an enhancer, specifically for the P1 promoter activity in T cells.

### NFATc1 activity prevents experimentally induced T-ALL

At the DN3 stage, though pTCR signalling is vital for proliferation and differentiation of thymocytes, it is equally crucial that the pTCR signals are switched off in the differentiated cells. Failure to do so will lead to the development of T-ALL, an aggressive form of leukaemia observed in both mouse and humans with constitutive Notch signalling^35^,^36^,^37^,^38^,^39^,^40^.

**Figure 7:**
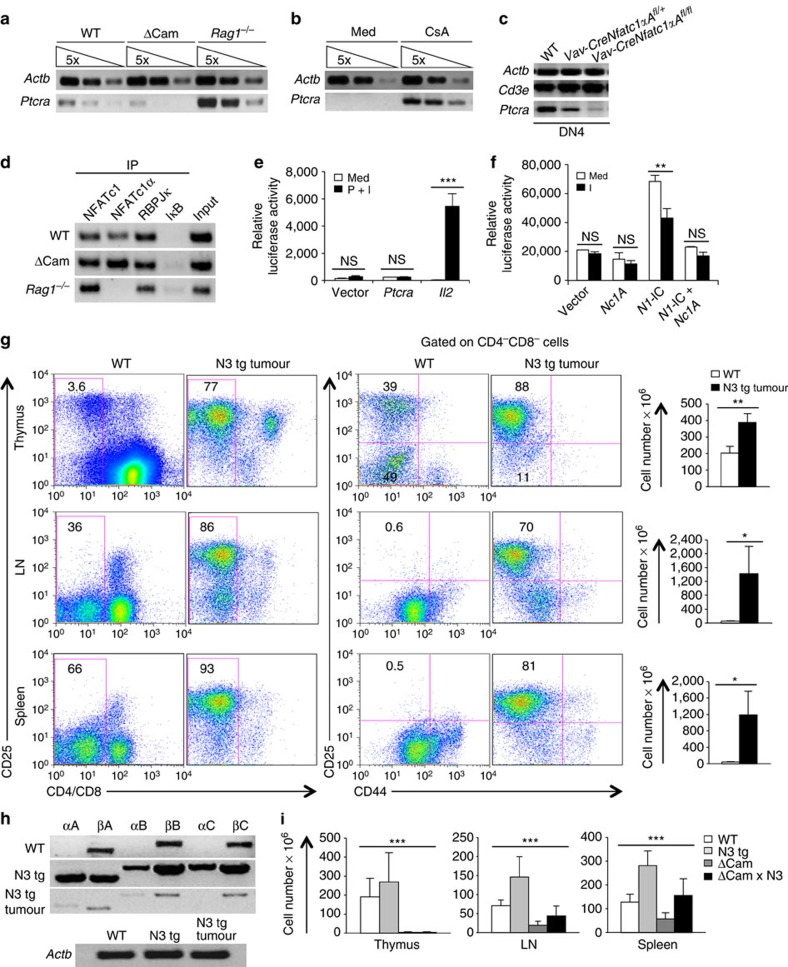
NFATc1α activity prevents T-ALL development. (**a**) RT–PCR analysis for *Ptcra* expression in DN3 cells from WT, ΔCam and *Rag1*^−/−^ mice. (**b**) *Ptcra* expression levels in CsA treated or untreated DN3 cells from ΔCam mice. (**c**) RT–PCR analysis for *Cd3e* and *Ptcra* expression in DN4 cells from WT, Vav-Cre *Nfatc1*αA^fl/+^ and Vav-Cre *Nfatc1*αA^fl/fl^ mice. (**d**) ChIP assays for *in vivo* NFATc1, NFATc1α, RβPJκ and IκBα binding at *Ptcra* promoter in WT DN cells or in *Rag1*^−/−^ and ΔCam DN3 cells. (**e**) Luciferase reporter assay depicting the influence of NFATc1α on *Ptcra* and *Il2* promoter activity in unstimulated or P+I stimulated EL-4 thymoma cells. (**f**) Effects of NFATc1α activity on Notch-induced *Ptcra* promoter transactivation in unstimulated or ionomycine (I) stimulated Jurkat T-ALL cells as revealed by luciferase reporter assays. (**g**) Flow cytometry profiles depicting the distribution of CD4^+^/CD8^+^ T-cell population (left panel), and CD4^−^CD8^−^CD44^-^CD25^+^ DN3 cells (right panel) in the thymus, LNs and spleen from mice with Notch3-induced T-ALL compared with WT littermate controls (*n*=7 per group). Histograms depict the cellularity in the thymus, LNs and in the spleen. (**h**) RT–PCR analysis of *Nfatc1* isoforms expression in N3-induced T-ALL cells compared with DN3 cells from WT or normal N3 tg mice. (**i**) Cellularity in the thymus, LNs and Spleen from WT, N3 tg, ΔCam and ΔCam × N3 double-tg mice (*n*=6 per group). Data are representative of three independent experiments and are shown as mean±s.d., ^***^P<0.0001, one-way analysis of variance. ^**^P=0.0039 or 0.0049 and *P=0.0317 or 0.0207, unpaired *t*-test.

We have previously shown that T-cell development in ΔCam mice is severely blocked at DN3 stage due to a defect in pTCR formation^9^. Further analysis revealed a strong downregulation in *Ptcra* (pTα) expression (Fig. 7a), in ΔCam DN3 cells. *Ptcra* expression is regulated by Notch signalling^41^, and accordingly, in N3 tg T cells pTα expression is not extinguished^38^. The negative effect of NFATc1α on *Ptcra* expression in ΔCam DN3 cells suggests that *Nfatc1* P1 activity might be involved in downregulating pTα expression once the cells have received pTCR signals. Inhibition of NFATc1 activity by CsA treatment restored *Ptcra* expression in ΔCam DN3 cells (Fig. 7b), further strengthening the possibility that NFATc1α suppresses *Ptcra* expression. This was confirmed by a dose-dependent suppression of *Ptcra* expression in DN4 cells in *Vav*-Cre*Nfatc1*α*A*^fl/+^ and *Vav*-Cre*Nfatc1*α*A*^fl/fl^ mice compared with WT cells (Fig. 7c). Further, ChIP assays confirmed NFATc1α binding at the *Ptcra* promoter *in vivo* in WT DN and ΔCam DN3 cells (Fig. 7d). Also, in reporter assays NFATc1α failed to induce *Ptcra* promoter activity whereas, it efficiently activated the murine *Il2* promoter (Fig. 7e). However, in human T-ALL cell line Jurkat, in reporter assays we observed a strong downregulation in Notch-induced *Ptcra* promoter activity by NFATc1α (Fig. 7f). Thus, our observations suggest that *Nfatc1* P1 activity critically influences Notch-pTCR signalling, necessary for normal T-cell development.

If this is the case, modulation of *Nfatc1* P1 activity might help to prevent T-ALL pathogenesis. We used N3-induced T-ALL as a model to check the role of NFATc1 in T-ALL development. N3 tg mice developed severe T-ALL with CD4^−^CD8^−^CD44^−^CD25^+^ DN3 cells accumulating in the thymus and peripheral lymphoid organs (Fig. 7g). As a result of tumorigenic transformation the differentiation of DN3 cells to T cells was drastically blocked in N3 tg tumour mice (Fig. 7g). Interestingly, *Nfatc1* expression in these N3-induced T-ALL cells was strongly downregulated compared with WT DN3 cells (Fig. 7h). We hypothesized that if the suppressed *Nfatc1* expression is the ‘cause' and not the ‘effect' of T-ALL, then restoring NFATc1 activity in N3 tg mice will help prevent leukemogenesis. To check this possibility *in vivo*, we crossed ΔCam mice with N3 tg mice to generate ΔCam × N3 double-tg mice. Surprisingly, in contrast to N3 tg mice, none of the ΔCam × N3 double-tg mice analysed, showed any sign of T-ALL (Fig. 7i). Rather, the double-tg mice showed a thymic phenotype similar to that observed in ΔCam mice (Fig. 7i; Supplementary Fig. 8a). Importantly, in ΔCam × N3 double-tg mice even though thymocytes were accumulated at the DN3 stage (Supplementary Fig. 8b), still they were not tumorigenic suggesting a strong tumour-preventing activity of NFATc1 in this experimental model.

## Discussion

We have shown previously the indispensability of NFATc1 activity in early thymocyte development^2^. Here we have delineated the signals that control *Nfatc1* expression initiated from two distinct promoters in a thymocyte developmental stage-specific manner and, how NFATc1 activity simultaneously facilitates the T-cell fate of the thymocytes, and prevents the pathogenesis of T-ALL. The finding that NFATc1 expression directed from only P2 promoter at the pTCR-negative stages and from both P1 and P2 promoters at the pTCR-positive stages establish the differential threshold of NFATc1 activity required for thymocyte differentiation. Once the DN3 cells receive pTCR signals, they are irreversibly committed towards T-lineage only. Hence, the NFATc1α activity at the pTCR-positive stages is the key to enhance the threshold of total NFATc1 activity through which T-lineage commitment is established on pTCR signals. Thus, the switch from ‘NFATc1β only' to both ‘NFATc1α and β' at the pTCR-positive DN3 cells is absolutely necessary for T-lineage commitment in the thymus. In this regard our observation that NFATc1 activity specifically suppresses the B-lineage plasticity of the immature thymocytes provides an important clue as to how activities of various transcription factors stabilize T-cell fate during development. Importantly, the characterization of a novel enhancer element specifically regulating the P1 promoter activities holds immense significance in the context of T-lineage commitment. From our observations, it appears that epigenetically E2 element is in an open conformation at the DN3 stage, but it is the specific transcription factor occupancy, which is critical in inducing P1 promoter activity at the pTCR-positive stage. As we have shown earlier that NFATc1 nuclear levels increases from DN1 to DN3 stage thus, most likely making it possible to bind to the E2 element and autoregulates its own expression by inducing P1 promoter activity. The elucidation of the signalling pathways regulating *Nfatc1* gene regulation will help to better understand T-cell development and will provide insight for therapeutic manipulations of NFATc1 activity to achieve immune reconstitution in situations like T-cell lymphopenia (Supplementary Fig. 8c).

The loss of *Nfatc1* expression in *in vitro* cultures though quite intriguing was not due to an enhanced cell death of the immature thymocytes (Supplementary Fig. 4a). Rather, because of very small number of sorted cells their density was too low in the *in vitro* cultures, which led not only to a lack of interaction between thymocytes and the thymic stroma, but it also resulted in a loss of interaction among thymocytes themselves. In the normal thymus, millions of thymocytes are very densely packed into the available space, which facilitates both thymocyte-stroma and also thymocyte–thymocyte interactions. In our opinion both types of interactions provide the signals essential for *Nfatc1* expression. In line with this *Nfatc1* expression was not lost when thymocytes were cultured *in vitro* at a higher density.

We have previously reported a severe T-cell lymphopenia in mice lacking NFATc1 activity in the early thymocytes^2^. Here we show overexpression of NFATc1 also results in T-cell lymphopenia (Fig. 3a-c; Supplementary Fig. 8c), indicating a threshold level of NFATc1 activity is critical for T-cell development. The increase in total NFATc1 activity in the *Vav*-Cre*Nfatc1*αA^fl/fl^ mice not only prevented the differentiation of DN3 cells to DN4 stage (Fig. 3d) but it also severely affected several key T-cell-specific genes expression. The dose effect of NFATc1, not only on the DN thymocyte development, but also on the DP to SP differentiation is clearly reflected in the cellularity of the thymus, LNs and spleen in the *Vav*-Cre*Nfatc1*αA^fl/fl^ mice compared with WT mice (Fig. 3b,c) underlining the influence of NFATc1 activity on thymic T-cell development.

Our results from ΔCam × N3 double-tg mice show the tumour suppressor activity of NFATc1 in the experimental setting of hyperactive Notch, as it prevented T-ALL development despite these mice having constitutive Notch3 signalling. *In vivo*, once the DN3 cells receive pTCR signals they undergo a limited cell cycle and differentiate to DN4 stage (Supplementary Fig. 8c). Unless the pTCR signalling is switched off, it will lead to uncontrolled proliferation resulting in T-ALL development (Supplementary Fig. 8c). To prevent pTCR formation, *Ptcra* expression needs to be silenced. Here we show that NFATc1α activity suppresses the *Ptcra* expression (Fig. 7a–c,e,f). As *Nfatc1* P1 activity is initiated only at the pTCR-positive thymocytes and pTCR signals induce NFATc1 activation^42^, this is most likely the natural-feedback mechanism to suppress pTCR expression and thereby prevent T-ALL development once the cells have received optimal pTCR signals (Supplementary Fig. 8c). However, loss of function mutations involving NFAT has not been reported in spontaneous cases of either human or murine T-ALL; thus, the tumour suppressive activity of NFATc1 may be specific to the background of the additional oncogenic events. Altogether, our study shows that a threshold level of NFATc1 activity is critical for T-cell development. Any alteration, such as above or below threshold levels of NFATc1 activity will push the delicate balance towards an unwanted extreme, either resulting in T-cell lymphopenia or, likely in combination with other oncogenic events, leading towards the development of leukaemia (Supplementary Fig. 8c).

## Methods

### Mice

C57BL/6 WT, calcineurin tg (ΔCam), *Rag1*^−/−^, *Notch3* tg (N3 tg), Cam × N3 tg, *Nfatc1* P2^fl/fl^, *Vav*-Cre*Nfatc1P2*^fl/+^, *Vav*-Cre*Nfatc1P2*^fl/fl^, *R26-caNfatc1aA-Stop*^fl/fl^ (*Nfatc1*αA^fl/fl^), *Vav*-Cre*Nfatc1*αA^fl/+^ and *Vav*-Cre*Nfatc1*αA^fl/fl^, *Nfatc1-eGfp-Bac* tg, *Nfatc1-eGfp-Bac*-*ΔE2* tg, *Vav-*Cre*Nfatc1*^fl/fl^ and *Vav-*Cre*Nfatc1αΑ*^fl/fl^*Nfatc1*^fl/fl^ mice of 3–8 weeks age were used in this study. Double-mutant mice such as, *Vav*-Cre*Nfatc1P2*^fl/fl^, *Vav*-Cre*Nfatc1*αA^fl/fl^ and *Vav-*Cre*Nfatc1*^fl/fl^ mice were generated by breeding corresponding floxed mice with *Vav-*Cre tg mice, or by breeding ΔCam with N3 tg (ΔCam × N3) mice, and the triple-mutant *Vav-*Cre*Nfatc1αΑ*^fl/fl^*Nfatc1*^fl/fl^ mice were generated by crossing *Vav*-Cre*Nfatc1*αA^fl/fl^ mice with *Nfatc1*^fl/fl^ mice. All mice were on BL/6 background and were maintained in the animal facility of the Institute of Pathology or in the central animal facility (ZEMM) of University of Wuerzburg, according to the institutional guidelines.

### Generation of *Nfatc1* P2^fl/fl^ mice

To generate the *Nfatc1* P2 promoter floxed allele, the 5′ end of the murine NFATc1 cDNA was used to isolate genomic cosmid clones of the *Nfatc1* gene. The 8.5 kb XbaI fragment containing the *Nfatc1* P2 promoter was identified in DNase I hypersensitive site analysis. The blunt ended left (XbaI to XhoI) and right (XhoI to XbaI) arms were subcloned into the SmaI site of pGL3 basic vector. To incorporate the loxP sites into the left or right arm, loxP sites cut out from the pBS112 SX vector were inserted into the NdeI (448 bp), Eco72I (5,020 bp) and NdeI (7536, bp) sites, respectively. The pTKNeoloxP plasmid containing the neomycin (neo) gene for positive selection and thymidine kinase gene for negative selection was used as basic vector in constructing the targeting vector. Embryonic stem cell clones positive for the targeted P2 floxed allele were selected and were used to generate the chimeric *Nfatc1* P2^fl/fl^ mice by blastocyst injection method. Genomic tail DNA from the chimeric mice was analysed by long-distance PCR to confirm the genomic integration of the floxed *Nfatc1* P2 allele, and mice were bred with C57BL/6 mice for several generations. WT, *Nfatc1* P2^fl/+^ and *Nfatc1* P2^fl/fl^ mice were genotyped using the following primers: 5′-TCTCCACCTGACTTTCTGTTCC-3′ (forward) and rev: 5′-CTCTTCCCAATGGTTGTCTCTC-3′ (reverse).

### Generation of *Nfatc1*-*eGfp-Bac*-*ΔE2* tg mice

*Nfatc1-eGfp-Bac*-*ΔE2* construct was generated by deletion of a 1-kb fragment of the E2 element from the original *Nfatc1-eGfp-Bac* transgene cassette^17^. The BAC RP23-361H16 containing the murine *Nfatc1* gene (mm9 chr.18, 80.779.051-80.993617, 214 kb) was further modified using bacterial homologues recombination to delete 1,000 bp from the intron 10 (mm9 chr.18, 80.809.298–80.808.298, ΔE2). Two homology arms flanking the region of interest were generated by PCR using the primers 5′-targ-box-E2_for: 5′-AAGGCGCGCCAAGAGGACCGGAACTCTGTG-3′, 5′targ-box-E2_rev: 5′-GCAGTGTCTATGGCTGTGCAAATCACCTG-3′, 3′targ-box-E2_for: 5′-TGCACAGCCATAGACACTGCAAGTCAGGG-3′, and 3′targ-box-E2_rev: 5′-AAGCGGCCGCCTCCACACACACACATATCC-3′. In a second PCR, the primers 5′targ-box-E2_for and 3′targ-box-E2_rev amplified a fusion product that was cloned as an AscI and NotI fragment into the shuttle vector pLD53.RecA. After homologous recombination the linearized BAC DNA was microinjected into blastocysts. Mice tail biopsies were tested by PCR for BAC integration and for deletion of the E2 element with the primers mNc1-DE2-for: 5′-CATACAAACCCACAAGTGACCA-3′ and mNc1-DE2-rev: 5′-GAGCCCTACTGAGAGATGGGAA-3′ (522 bp). The 5′-end of integrated BAC DNA was detected with the primer pair 5′BAC(T7) for: 5′-GGTCCATCCTTTTGTCTCA-3′ and 3′e3.6(T7) rev: 5′-CGAGCTTGACATTGTAGGA-3′ (512 bp), and the 3′ end with the primers 5′e3.6(SP6) for: 5′-CGTCGACATTTAGGTGACA-3′ and 3′BAC (SP6) rev: 5′-CCATCGTTCCCTGACTCA-3′ (439 bp).

### Antibodies and flow cytometry

All Abs used were purchased from BD Pharmingen or from eBioscience: anti-CD4 (GK1.5), anti-CD8α (53-6.7), anti-CD25 (PC61), anti-CD44 (IM7), anti-CD2 (RM2-5), anti-CD3ɛ (145-2C11), anti-CD5 (53-7.3), anti-B220 (RA3-6B2), anti-CD11a (2D7), anti-CD11c (N418), anti-CD18 (C71/16), anti-CD24 (M1/69), anti-CD48 (HM48-1), anti-CD49d (R1-2), anti-CD62L (MEL-14), anti-TCRβ (H57-597), anti-c-Kit (2B8), anti-NK1.1 (PK136), anti-F4/80 (BM8), anti-Gr1 (RB6-8C5), anti-Ter119 (TER-119), anti-c-Kit (2B8), Annexin V, anti-Bcl-2 (3F11) and Hamster IgG1 Isotype control (A19-3). Biotinylated antibodies were revealed with Streptavidin-Allophycocyanin (APC) or Phycoerythrin-Cy5 (PE-Cy5). For surface staining all primary and secondary antibodies were diluted at 1:300 in PBS containing 0.1% bovine serum albumin (BSA) and 0.01% sodium azide. Cell death analysis of DN4 cells cultured overnight in medium was performed by Annexin V (1:100) staining following manufacturer's instruction (BD Pharmingen). Flow cytometry was performed according to standard protocol using FACSCalibur and CellQuest or FlowJo software.

### Cell sorting

For FACS sorting of DN1 to DN4 cells, DN thymocytes were enriched by treatment of total thymocytes with biotinylated Abs against CD4 (GK1.5), CD8 (TiB105), NK1.1 (1D4), CD19 (1D3) and MHC class II (2G9) followed by negative selection using Anti-Biotin MicroBeads (Miltenyi Biotec). Purity of DN cells was always 90–95% as verified by FACS analysis. Enriched DN thymocytes were further stained with APC-conjugated CD4 and CD8, PE-conjugated CD25 and FITC-conjugated CD44 Abs to sort the DN1–DN4 cells gating on the CD4^−^CD8^−^population using a FACSVantage or FACSAria (BD Biosciences) flow cytometer.

### Intracellular staining

Total thymoctyes of 1 × 10^6^ were first surface stained for CD4, CD8, and CD25 molecules. Afterwards, cells were fixed in 1% paraformaldehyde (15 min at room temperature) and permeabilized in PBA (1 × PBS, 5% BSA and 0.02% NaN_3_) containing 0.5% saponin (10 min at room temperature). After permeabilization cells were incubated with FITC conjugated mAbs against TCRβ (1:100), CD3ɛ (1:100), Bcl-2 (1:50) or Hamster IgG1 Isotype control (1:5) diluted in PBA containing 0.5% saponin for 60 min at room temperature. Cells were washed twice in 1 ml of PBA/0.5% saponin, once with PBS/0.1% BSA/0.01% azide and analysed by FACS.

### Immunofluorescence staining and ChIP assays

Sorted WT DN1+DN2+DN3 or DN4 cells were fixed in 0.5% formaldehyde, followed by cytospin on to glass slides at 300 r.p.m. for 3 min. Cells were incubated with anti-NFATc1α (ImmunoGlobe; IG-457), followed by Alexa Fluor 555–conjugated donkey anti-rabbit IgG (A-31572; Molecular Probes) and DAPI (4,6-diamidino-2-phenylindole; Molecular Probes). Image acquisition and analysis were done with a TCS SP2 Leica confocal microscope and software.

ChIP assays on WT DN thymocytes, or *Rag1*^−/−^ and ΔCam DN3 cells with anti-NFATc1 (Santacruz; sc-7294), anti-NFATc1α, antiRBPJκ (Cell Signaling; 5313) and anti-IκB-α (Santa Cruz; sc-371) were done as described previously^2^. Briefly, 1 × 10^7^ freshly isolated DN thymocytes from WT, or DN3 cells from *Rag1*^−/−^ and ΔCam tg mice were cross-linked using 1% formaldehyde. Isolated nuclei were sonicated so that the average length of chromosomal DNA became 500–750 bp. Chromatin solution was precleared with Sepharose G, and chromatin was immunoprecipitated by incubating with 6–8 μg of each antibody mentioned above for overnight at 4 °C. Immune complexes were collected on protein G-Sepharose beads and immunoprecipitates were eluted with 1% SDS, 50 mM NaHCO_3_. After reversal of cross-links and deproteination, purified DNA was used for amplification of the *Ptcra* promoter region bound to NFATc1, NFATc1α, and RBPJκ with the following primers: 5′- AGGGAACAGAATTCAAGGCTG-3′ (forward) and 5′-ATCTTCTTGCTCTAAATCTCC-3′ (reverse).

### ChIP-Seq and RNA-Seq analysis

ChIP-seq^43^ and Small RNA-seq^8^ were performed as described previously using same standards and quality controls.

### ChIP-Seq

Briefly, DN cells were isolated from Bl.6 *Rag2*^−/−^ mice and cross-linked with 1% formaldehyde. Histone ChIPs were performed by using cross-linked nuclear extracts from 5 million cells with 2 μg specific antibodies and 20 μl Dynabeads Protein G suspension (Life Technologies, USA), whereas 50 million cells were used for Pol II ChIPs. Antibodies used are described in Koch *et al.*, with the exception of H3K27ac (ab4729; Abcam). DNA was extracted from immunoprecipitated chromatin and was used to prepare sequencing library using TrueSeq ChIP-seq Library Preparation Kit (Illumina Inc., USA) according to manufacturer's instructions, and sequenced on a Genome Analyzer II sequencing platform (Illumina Inc., USA). Data processing, including input subtraction and wig files generation was performed as previously described^43^.

### Small RNA-Seq

Total RNA was extracted from DN cells isolated from *Rag2*^−/−^ cells using TriZol Reagent (Life Technologies, USA). Total RNA of 10 μg was fractionated by TBE-Urea PAGE to isolate RNA fragments ranging from 15 nucleotides to 70 nucleotides length. These size-selected small RNAs were used to prepare sequencing library using Small RNA-Seq Library Preparation Kit (Illumina Inc., USA) according to manufacturer's instructions. Libraries were then sequenced on a Genome Analyzer II sequencing platform (Illumina Inc., USA). The RNA-Seq data used is accessible in gene omnibus under the accession number GSE44578.

### OP9-DL1 co-culture assays and *in vitro* cultures

4 × 10^4^ sorted WT or *Vav*-Cre*Nfatc1*αA^fl/fl^ DN1- DN4 cells were co-cultured on monolayers of OP9 stromal cells expressing the Notch ligand delta-like 1 (OP9-DL1) in X-vivo 20 medium supplemented with rhFLT3 ligand (5 ng ml^−1^) and rhIL-7 (1 ng ml^−1^) for 4 days. Subsequently, thymocytes were analysed for differentiation into DP cells by flow cytometry. *In vitro* cultures of sorted DN1–DN4 cell populations or CD4^+^ T cells were performed either in medium (RPMI-1640, 10% FCS) only or with CsA (100 ng ml^−1^), 8-CPT-cAMP (50 μM), Forskolin (Fsk; 100 μM), CD48 (5 or 0.5 μg ml^−1^), PMA (100 ng ml^−1^), Fibronectin (Fibr; 1 μg ml^−1^) supplemented with rhIL-7 (10 ng ml^−1^).

### Semiquantitative RT–PCR

For RT–PCR, cDNA was synthesized from cells either freshly isolated or cultured as indicated in each case using Miltenyi Biotech cDNA synthesis kit. To check CD2-CD48 signalling-induced *Nfatc1* expression, 1 × 10^6^ sorted WT DN3 cells were cultured in cRPMI-1640 medium (10% fetal calf serum) supplemented with 10 ng ml^−1^ rhIL-7. Cells were either left unstimulated or stimulated with plate bound CD48 antibodies (5 μg), PMA (100 ng) or with CD48+PMA for 12 h at 37 °C. Similarly, 8 × 10^6^ CD4^+^ T cells from lymph nodes of WT mice were left unstimulated or stimulated with plate bound Fibronectin (1 μg), CD48 (0.5 μg) or Fibronectin+CD48 for 24 h at 37 °C. Semiquantitative RT–PCR was performed to check the levels of expression of indicated genes. β*-actin* expression levels were used as control. RT–PCRs to check the expression of six *Nfatc1* isoform were performed using the Fermentas Long distance PCR Kit (Cat. # K0181). Primer sequences are available (Supplementary Table 1) in the Supplemental Information online.

### Methylation analysis

Genomic DNA was isolated from FACS sorted WT DN3 and DN4 cells following standard protocol. Sodium bisulphite treatment of genomic DNA was performed using ZYMO Research EZ DNA Methylation Gold kit (Cat. # D5005) according to manufacturer's instructions. All PCR were performed on MWG AG Biotech Primus 96 plus thermocyclers (Ebersberg, Germany) in a final volume of 25 μl including 1 × PCR Master Mix (Thermo Scientific, Cat. # K0171), 10 pmol each of forward and reverse primers (*Nfatc1* P1d forward: (-1297 -818) 5′-GGAGATTTGAAAGAGAAAAA-3′ and *Nfatc1* P1d reverse: (-1297 -818) 5′-AAARCTACTCTCCCTTTTAAT-3′), and 10 ng of bisulphite-treated genomic DNA. The following amplification protocol was used: 95 °C for 10 min and 40 cycles of 95 °C for 1 min, 52 °C for 45 s, and 72 °C for 1 min, and a final amplification step of 72 °C for 10 min. PCR products were eluted and purified using innuPREP DOUBLEpure kit (Analytik Jena AG, Cat. # 845-KS-5050050) and cloned into a pGEM T-Easy Vector (Promega, Cat. # A1360) according to manufacturer's protocol. *E. coli* (Top-F) were transformed with plasmid ligates and were selected by amp^R^ on Lurient-Broth Agar plates. Colonies expressing the full-size amplificate were detected by blue-white selection and DNA was prepared according to standard protocols. Positive clones were further determined by restriction digestion (EcoR*I*) and subsequently sequenced. PCRs were performed with primers for T7 and SP6 (Sigma-Aldrich) in both 5′ and 3′ direction using Big Dye Terminator v3.1 Cycle Sequencing Kit (Applied Biosystems, Product P/N 4336935). Sequence data was generated with an ABI Prism 3130xl, and analysed with BiQ Analyzer v2.0 (Christoph Bock).

### Immunoblotting

Positively selected LN CD4^+^ T cells (2 × 10^7^) from *Nfatc1-eGfp-Bac* tg or *Nfatc1-eGfp-Bac*-*ΔE2* tg reporter mice were left unstimulated or stimulated with aCD3 (3 μg ml^−1^) plus aCD28 (5 μg ml^−1^) for 48 h. Subsequently, whole-cell protein extracts were prepared and 20 μg protein from each sample was subjected to immunoblotting in 12% polyacrylamide gels. Proteins were transferred to a nitrocellulose membrane followed by immunodetection of GFP with Abs directed against GFP (Santacruz; sc-9996), or NFATc1α with Abs raised against the α-peptide of NFATc1 (ImmunoGlobe; IG-457). Images in Fig. 6d are cropped for presentation excluding the lanes 3 and 6, which represents B-cell extracts. Full-size images from original scan with molecular weight marker are presented in Supplementary Fig. 7.

### Luciferase reporter assays

One microgram of a murine *Ptcra* luciferase reporter construct containing 400 bp enhancer region (−4,400 to 4,000 bp) and 1.1 kb DNA fragment spanning murine *Ptcra* promoter region (−1034 to +66 bp), or 1 μg of a luciferase reporter construct containing 293 bp DNA fragment spanning murine proximal *Il2* promoter region was co-transfected along with 4 μg control vectors into EL-4 thymoma cells by DEAE/Dextran method. Thirty six hours post-transfection cells were left unstimulated or stimulated with PMA plus Ionomycin (100 ng ml^−1^ each, Calbiochem) for 12 h.

Afterwards, luciferase activity representing the *Ptcra* promoter transactivation was measured using a MicroLumat LB 96P (EG&G Berthold) luminometer. For *Nfatc1* P1 and P2 promoter constructs, a 1,430 bp (-1155 to +275) DNA fragment in the region around exon 1, and a 1260, bp (-1205 to +55) DNA fragment in the region around exon 2, respectively, were cloned as NheI/XhoI-digested PCR products into the pGl3basic vector. The potential enhancer elements E1–E4 were cloned 3′ of the luciferase reporter gene as SalI-digested PCR products. Mutations of potential NFAT, GATA and PU.1 binding sites in the E2 element were introduced by site-directed mutagenesis. The details about the NFATc1 (Nc1A) and Notch1 intracellular domain (N1-IC) expression constructs have been described previously^5^,^44^. To assess the effects of NFATc1 activity on Notch-induced *Ptcra* promoter activation, 1 × 10^6^ Jurkat cells were transfected by the ViaFect Transfection Reagent (Promega, Wisconsin, USA) with 5 μg DNA containing all necessary constructs according to the manufacturer's protocol. Twenty four hours later fresh medium was added and the cells were divided into untreated or ionomycin (I) treated samples. After further 16 h cells were collected to estimate luciferase activity.

### Data availability

ChIP-Seq (accession numbers GSE80138 and GSE55635) and RNA-Seq (accession number GSE44578) data are available in the gene omnibus database. All other data supporting the findings of this study are available with the article, and can also be obtained from the authors.

## Additional information

**How to cite this article**: Klein-Hessling, S. *et al.* A threshold level of NFATc1 activity facilitates thymocyte differentiation and opposes notch-driven leukaemia development. *Nat. Commun.* 7:11841 doi: 10.1038/ncomms11841 (2016).

## Supplementary Material

Supplementary Information Supplementary Figures 1 - 8 and Supplementary Table
