# Supplementary material for: A threshold level of NFATc1 activity facilitates thymocyte differentiation and opposes notch-driven leukaemia development
Source: Nat Commun. 2016 Jun 17;7:11841. doi: 10.1038/ncomms11841 (PMC4915031; doi:10.1038/ncomms11841)
Supplement: Supplementary Information — Supplementary Figures 1 - 8 and Supplementary Table [file ncomms11841-s1.pdf]

Supplementary Information

Supplementary Figure 1

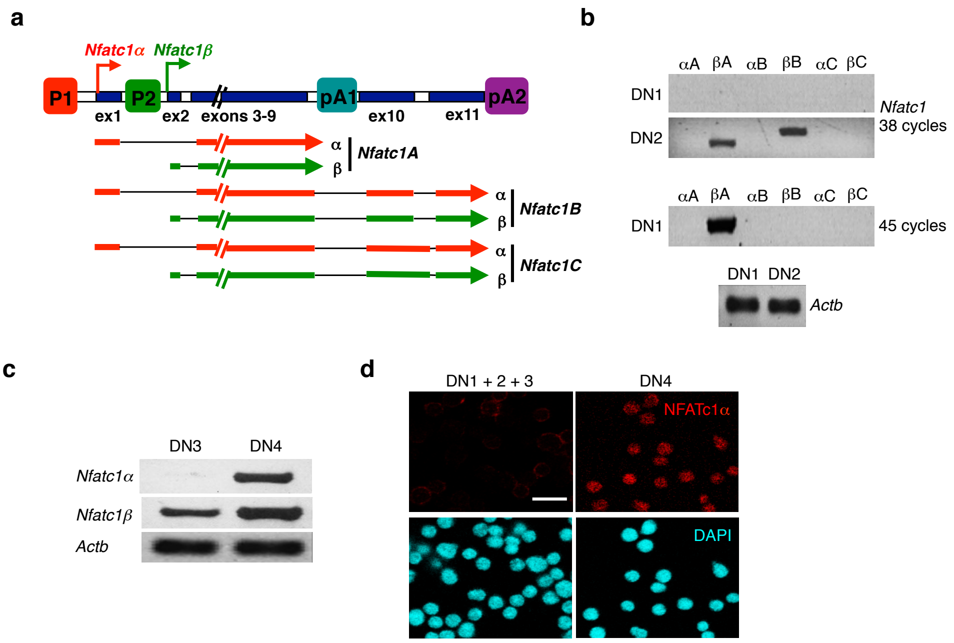

Supplementary Figure Legends

Supplementary Figure 1 Exclusive *Nfatc1* P2 promoter activity in pTCR-negative thymocytes (**a**) Schematic representation of the *Nfatc1* gene and the six *Nfatc1* isoforms derived from P1 and P2 promoters due to alternate splicing and usage of two different polyadenylation (pA) sites. (**b**) RT-PCR analysis of *Nfatc1* isoforms expression in sorted WT DN1 and DN2 cells. Only *Nfac1*β isoforms derived from the P2 promoter was detectable even with increased number of PCR cycles. (**c**) Analysis of total P1 or P2 promoter-derived *Nfatc1* transcripts in freshly isolated WT DN3 and DN4 cells. (**d**) Immunofluorescence analysis of NFATc1α levels in sorted WT pTCR-negative (DN1+2+3) and -positive (DN4) cells. Nuclear NFATc1α was confirmed with counter staining with DAPI. Scale bar, 10 μm.

Supplementary Figure 2

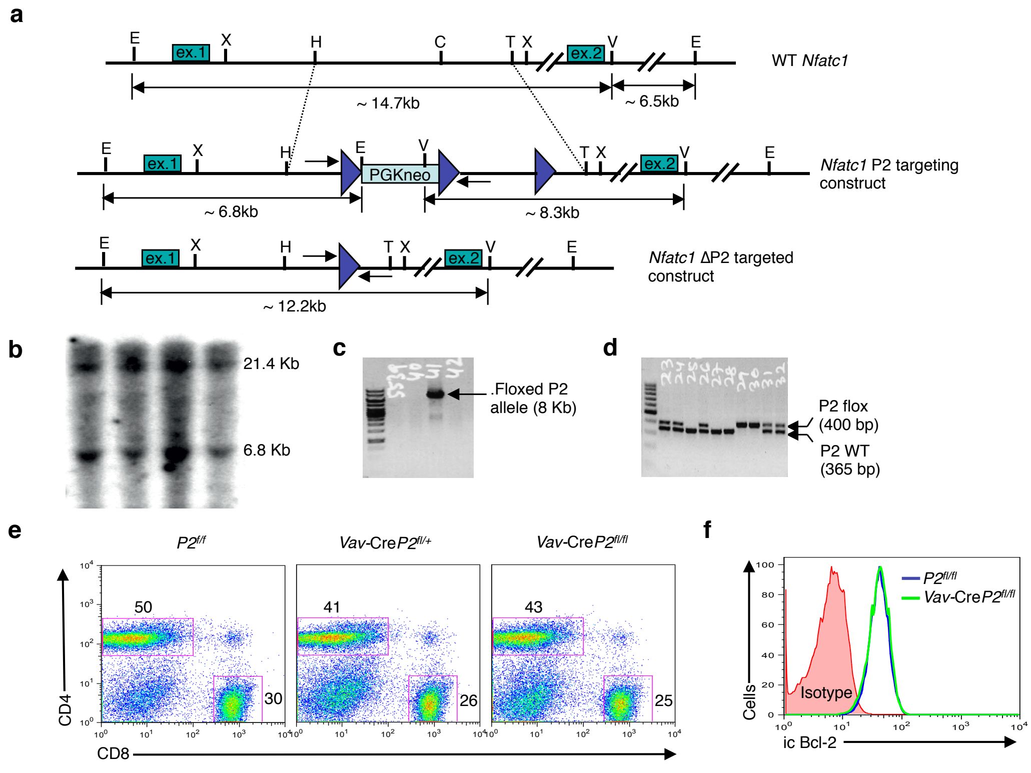

Supplementary Figure 2 Normal T cell development in *Nfatc1* P2 promoter-deficient mice (**a**) Gene targeting strategies to generate the targeting vector for generation of the *Nfatc1* P2 promoter floxed mice. Rectangles and triangles represent exon and flox sites respectively. Restriction enzyme sites are denoted in capital letters; *EcoRI* (E), *XbaI* (X), *XhoI* (H), *Eco72I* (C), *AatII* (T), *EcoRV* (V). (**b**) Southern blot to detect embryonic stem (ES) cell clones positive for the targeted P2 floxed allele. (**c**) Long distance PCR with genomic tail DNA to detect the integration of the floxed P2 targeting vector. (**d**) Genotyping PCR to detect WT (365 bp), heterozygous and homozygous (400 bp) P2 floxed mice using genomic tail DNA. (**e**) $CD4^+$ and $CD8^+$ T cells distribution in lymph nodes from WT, *Vav*-Cre*Nfatc1P2*$^{fl/+}$ and *Vav*-Cre*Nfatc1P2*$^{fl/fl}$ mice. Numbers inside each plot represent percent respective population. (**f**) Intracellular Bcl-2 levels in DN3 cells from WT (*P2*$^{fl/fl}$) and *Vav*-Cre*Nfatc1P2*$^{fl/fl}$ mice. Data are representative of 3 independent experiments (**e & f**), (n = 3 per group per experiment).

Supplementary Figure 3

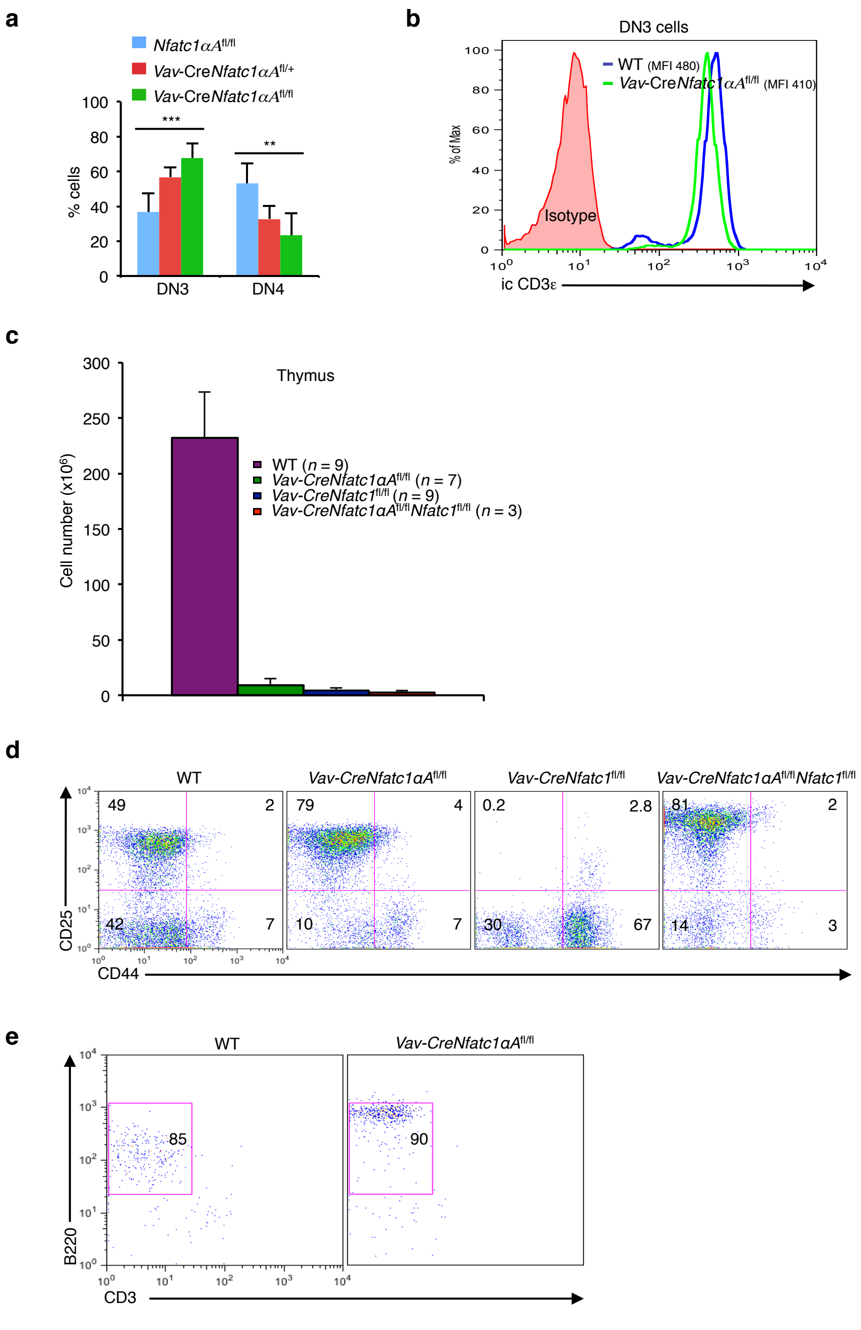

Supplementary Figure 3 Defective T cell development in *Vav*-Cre*R-26*-ca*Nfatc1αA*-*Stop*$^{fl/fl}$ (*Vav*-Cre*Nfatc1αA*$^{fl/fl}$) mice (**a**) Increase in $CD4^{-}CD8^{-}CD44^{-}CD25^{+}$ DN3 and decreased $CD4^{-}CD8^{-}CD44^{-}CD25^{-}$ DN4 cells in *Vav*-Cre*Nfatc1αA*$^{fl/fl}$ (*n* = 14) mice compared to WT (*n* = 6) or *Vav*-Cre*Nfatc1αA*$^{fl/+}$ (*n* = 24) littermates. (**b**) Flow cytometry profiles of intracellular CD3ε levels in DN3 thymocytes from *Vav*-Cre*Nfatc1αA*$^{fl/fl}$ mice compared to WT mice. (**c**) Thymic cellularity in the *Vav-CreNfatc1αA*$^{fl/fl}$*Nfatc1*$^{fl/fl}$ mice compared to that in WT, *Vav*-Cre*Nfatc1α*A$^{fl/fl}$ and in *Vav-CreNfatc1*$^{fl/fl}$ mice. *Vav-CreNfatc1αA*$^{fl/fl}$*Nfatc1*$^{fl/fl}$ mice only express NFATc1α from the knocked-in gene and lack NFATc1 expression from the endogenous gene. (**d**) Flow cytometry analysis of DN thymocytes from WT, *Vav*-Cre*Nfatc1α*A$^{fl/fl}$, *Vav-CreNfatc1*$^{fl/fl}$ and *Vav-CreNfatc1αA*$^{fl/fl}$*Nfatc1*$^{fl/fl}$ mice for the distribution of DN1 to DN4 cells. (**e**) Unimpaired B-lineage differentiation potential of *Vav*-Cre*Nfatc1α*A$^{fl/fl}$ DN1 cells. 4 x 10^4 sorted WT or *Vav*-Cre*Nfatc1α*A$^{fl/fl}$ DN1 cells were co-cultured on monolayers of OP9 stromal cells in X-vivo 20 medium supplemented with rhFLT3 ligand (5 ng/ml) and rhIL-7 (1 ng/ml) for 6 days. Subsequently, the cells were analyzed for differentiation into B ($B220^{+}$) or T ($CD3^{+}$) lineage by flow cytometry. Numbers within each plot represent percent respective population. Data are representative of three independent experiments and are shown as mean ± s.d., $^{***}P = 0.0006$ and $^{**}P = 0.0036$, One-way ANOVA.

Supplementary Figure 4

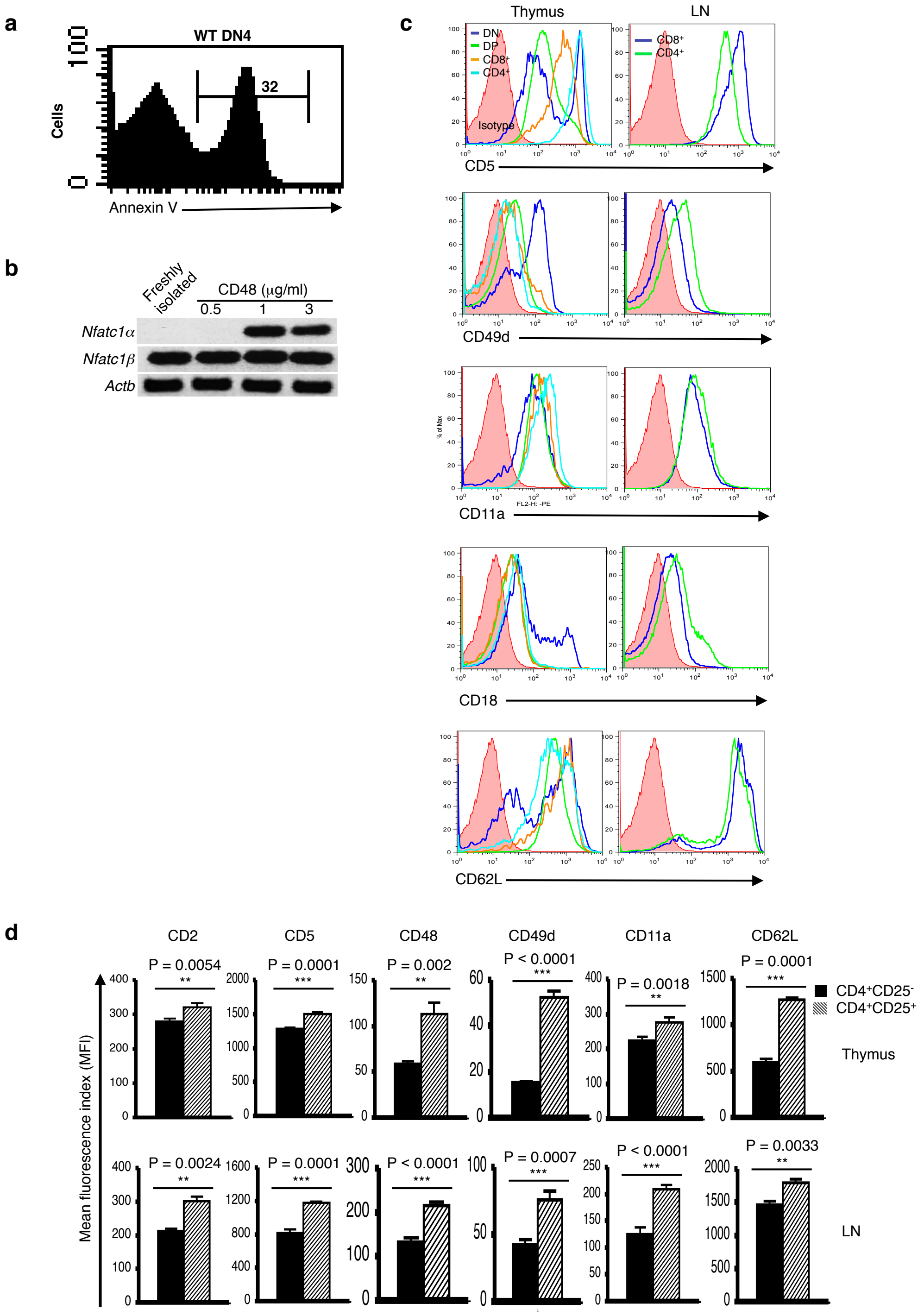

Supplementary Figure 4 Differential expression of various integrins in thymocyte subsets and in T cells (**a**) Annexin V analysis to discriminate live and dead cells in WT DN4 cells cultured in complete RPMI-1640 medium (10% FCS) for 12 h. (**b**) RT-PCR analysis for *Nfatc1*α and *Nfatc1*β expression in WT DN3 cells stimulated with indicated concentrations of CD48 Abs for 18 h. (**c**) Flow cytometry profiles of CD5, CD49d, CD11a, CD18 and CD62L expression on DN, DP, $CD4^+$ and $CD8^+$ SP T cells from thymus and in $CD4^+$, and $CD8^+$ T cells from LNs of WT mice. (**d**) Expression of various integrins on $CD4^+CD25^+$ regulatory T cells (T_{reg}) from thymus and LNs of WT mice (n = 4), compared to $CD4^+CD25^-$ effector T (T_{eff}) cells. Histograms depict mean fluorescence intensity (MFI) for indicated integrin. Data are representative of three independent experiments and are shown as mean ± s.d.,paired *t*-test.

Supplementary Figure 5

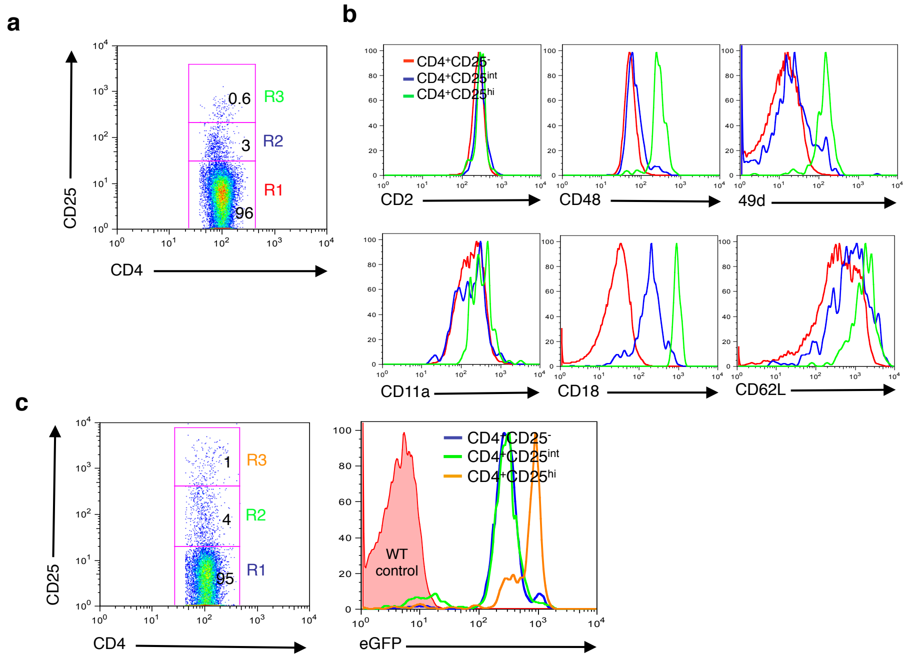

Supplementary Figure 5 The level of integrin expression correlates with that of *Nfatc1* expression (**a**) Dot plot showing the three distinct populations of thymic $CD4^+$ T cells; $CD4^+CD25^-$, $CD4^+CD25^{lo}$ and $CD4^+CD25^{hi}$ from WT mice based on CD25 expression. (**b**) Flow cytometry profiles of indicated integrin expression levels on thymic $CD4^+CD25^{hi}$ cells compared to $CD4^+CD25^{lo}$ and $CD4^+CD25^-$ cells from WT mice. (**c**) Flow cytometry profiles demonstrating the thymic $CD4^+CD25^{hi}$, $CD4^+CD25^{lo}$ and $CD4^+CD25^-$ cells, and their corresponding *Nfatc1* expression levels as measured by GFP expression in *Nfatc1-eGfp-Bac* tg reporter mice.

Supplementary Figure 6

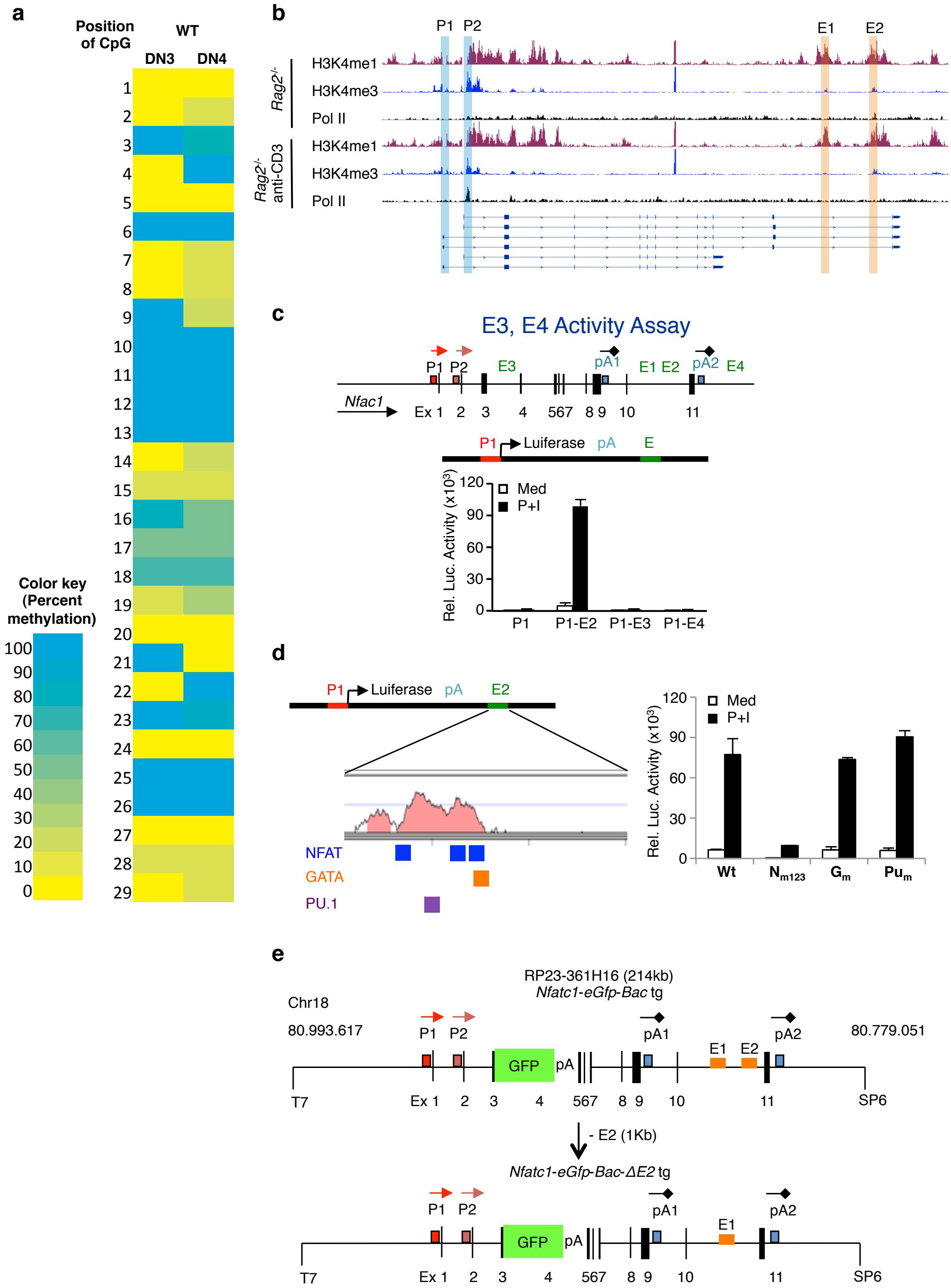

Supplementary Figure 6 DNA methylation status at the *Nfatc1* P 1 promoter in WT DN3 and DN4 cells (**a**) CpG methylation profiles of a 497 bp (-818 till -1297) segment of the murine *Nfatc1* P1 promoter region from WT pTCR-negative DN3 and pTCR-positive DN4 cells. The extent of methylation on individual cytosine residues is as indicated by the color code. (**b**) ChIP-Seq analysis of *Nfatc1* gene in aCD3 Abs stimulated *Rag2*$^{-/-}$ DN3 cells for epigenetic modifications at P1 and P2 promoter regions as well as at the putative regulatory elements compared to unstimulated cells. Blue rectangles represent the promoters, and the pink rectangles highlight the putative enhancer regions. (**c**) The positions of the putative regulatory elements E3 and E4 in the *Nfatc1* locus, and their influence on Nfatc1 P1 promoter activity as demonstrated by luciferase reporter assays in EL-4 thymoma cells. (**d**) Analysis of putative transcription factor binding motifs in the E2 element and the effects of mutation in individual motifs on *Nfatc1* P1 promoter inducibility as revealed by luciferase reporter assays in EL-4 cells. (**e**) Schematic diagram of the *Nfatc1-eGfp-Bac-ΔE2* transgene construct used to generate the reporter mice. A 1 kb region of the E2 element was deleted from the original *Nfatc1-eGfp-Bac* tg cassette to inactivate the enhancer function.

Supplementary Figure 7

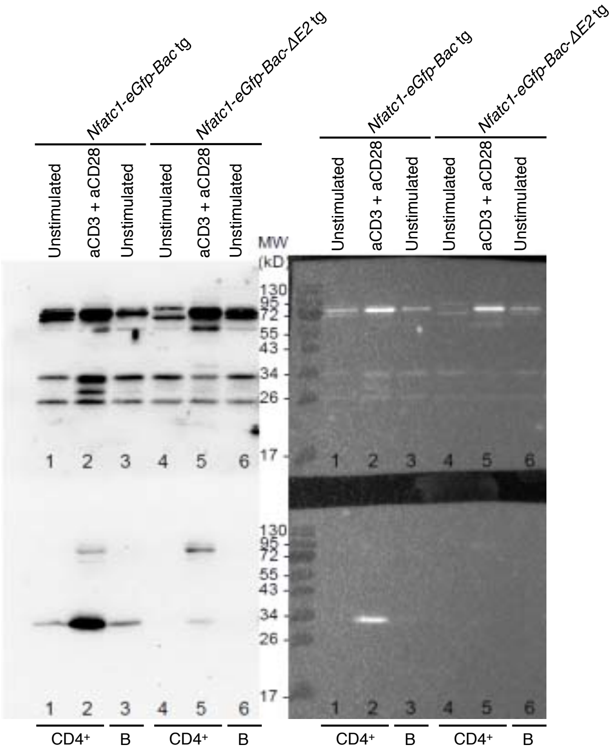

Supplementary Figure 7 *Nfatc1* P 1 promoter activity is regulated by the E2 enhancer element. Whole cell extracts from 2 x 10^7 positively selected LN $CD4^+$ T-cells either left unstimulated or stimulated with aCD3 + aCD28 Abs for 48 h, and freshly isolated $CD19^+$ splenic B cells were resolved in 12% polyacrylamide gels to detect GFP (upper panel) and NFATc1α (lower panel) expression in lymphocytes. Immunoblot images were recorded in a Vilber Lourmat Fusion-SL imager and data were analyzed using the Fusion Capt. V16 software. The original peroxidase signals (right panel) and the converted negative image (right panel) of the same membranes with molecular weight markers are shown. Protein extracts were prepared from *Nfatc1-eGfp-Bac* tg (lanes 1-3) and from *Nfatc1-eGfp-Bac-ΔE2* tg (lanes 4-6) lymphocytes as indicated, i.e. unstimulated LN $CD4^+$ T-cells (lanes 1, 4), 48h aCD3 + aCD28 stimulated LN $CD4^+$ T-cells (lanes 2, 5) and unstimulated $CD19^+$ B-cells (lanes 3, 6).

Supplementary Figure 8

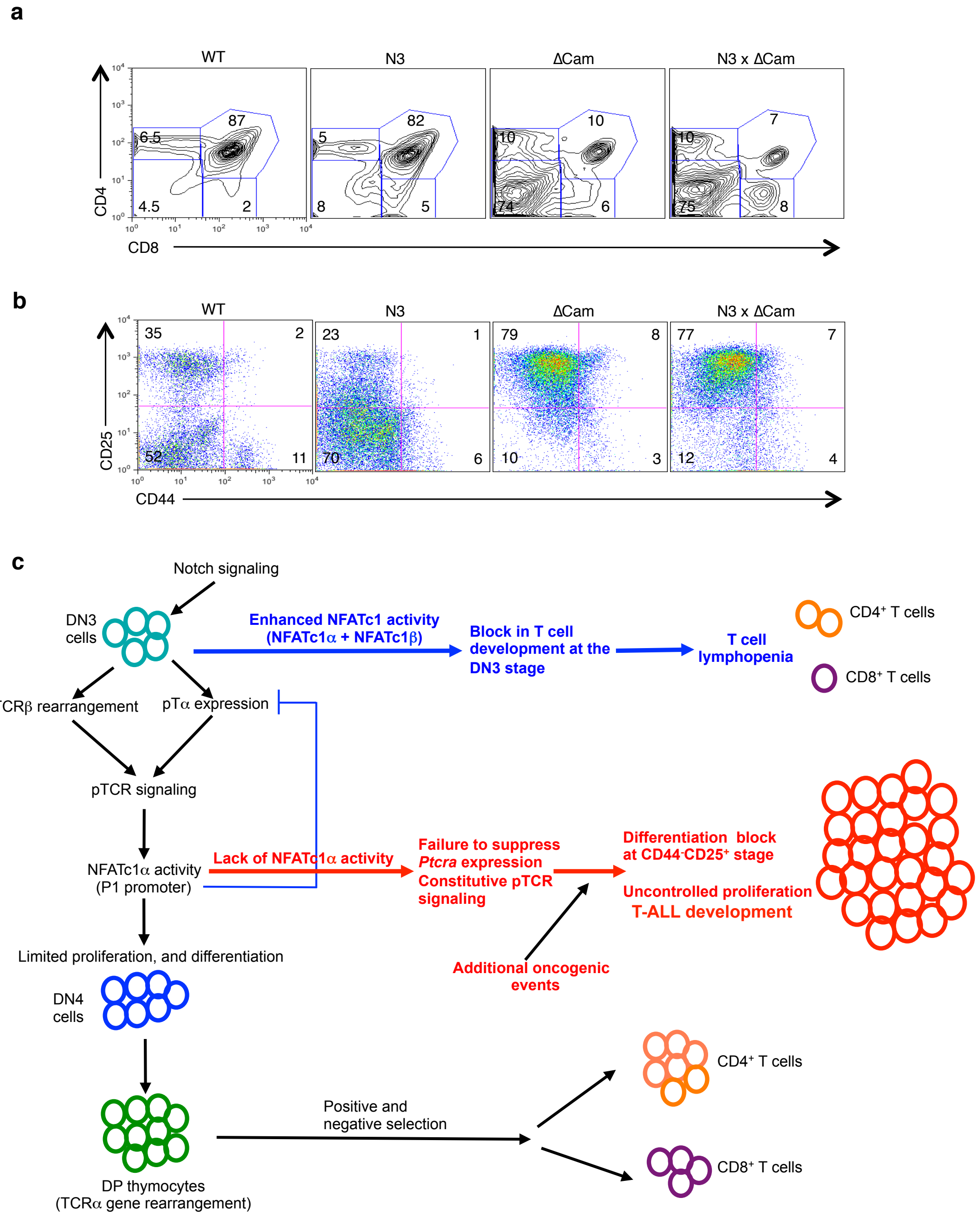

Supplementary Figure 8 NFATc1 activity plays an essential role in promoting T cell differentiation and in preventing Notch-induced T-ALL development (**a**) Flow cytometry profiles reveal the distribution of thymocyte subsets based on CD4 and CD8 expression in WT, N3 tg, ΔCam and N3 x ΔCam double tg mice (**b**) DN1-DN4 cells distribution within DN thymocytes based on CD44 and CD25 expression in indicated mice. Numbers inside each plot represent percent respective population. Data in (**a)** and (**b)** are representative of three independent experiments (n = 3 per group per experiment). (**c**) Scheme showing the influence of pTCR signaling-induced NFATc1α activity in facilitating T cell differentiation and in preventing T-ALL development. DN3 cells upon receiving Notch signals express the pTCR on their surface. Subsequently, pTCR signaling induces NFATc1α activation, which promotes a limited proliferation followed by differentiation of pTCR-positive DN3 cells to the DN4 stage. Simultaneously, NFATc1α activity suppresses the expression of *Ptcra* once the DN3 cells have received optimal pTCR signals. DN4 cells further differentiate to DP thymocytes, which through the process of positive and negative selection give rise to a normal repertoire of $CD4^+$ or $CD8^+$ SP T cells. Alterations to this physiological process such as, enhanced NFATc1 activity due to NFATc1α expression in pTCR-negative DN3 cells will lead to severe T cell lymphopenia, or lack of NFATc1α activity in pTCR-positive DN3 cells in combination with other oncogenic events will result in the development of leukemia (T-ALL) due to uncontrolled proliferation.

Supplementary Table 1

LIST OF RT-PCR PRIMERS

Gene	Primer Sequence	Product Size
Actb	For: 5'-CCAGGTCATCACTATTGGCAAGGA-3'<br>Rev: 5'-GAGCAGTAATCTCCTTCTGCATCC-3'	223 bp
Nfatc1α<br><br>*Nfatc1β*<br><br>*Nfatc1*A<br><br>*Nfatc1*B<br><br>*Nfatc1*C	For: 5'-ATGCCAAGCACCAGCTTTCCAGTCCCTT CC-3'<br>For: 5'-ATGACGGGGCTGGAGCAGGACCCGGAG TTC-3'<br>Rev: 5'-CCTCAGAGCTTAAGGTTAGAAAGACAG AGTTACC-3'<br>Rev: 5'-ACGTATGATCTCATTTACTGCGGCTGTA G-3'<br>Rev:5'-GGACAGCTACTGTTCAGATGTGGACTC AC-3'	2000 – 2500<br><br>bp
Nfatc1α:<br>(P1 activity)	For: 5'-GGGAGCGGAGAAACTTTGC-3'<br>Rev: 5'-GATCTCGATTCTCGGACTCTCC-3'	319 bp
Nfatc1β:<br>(P2 activity)	For: 5'-CGACTTCGATTTCCTCTTCGAG -3'<br>Rev: 5'- GATCTCGATTCTCGGACTCTCC-3'	311 bp
Nfatc1	For: 5'-GACTTCGATTTCCTCTTCGAGTTC-3'<br>Rev: 5'-CTCGATTCTCGGACTCTCCAG-3'	297 bp
Nfatc2	For: 5'-GGGTTCGGTGAGTGACAGTT-3'<br>Rev: 5'-CTCCTTGGCTGTTTGGGATA-3'	371 bp
Nfatc3	For: 5'-CCGATGACTACTGCAAACTGTGG-3'<br>Rev: 5'-TTTGAATACTTGGGCACTCAAAGG-3'	343 bp
Notch1	For: 5'-TTGACGTCACTCTCCTGTGC-3'<br>Rev: 5'-ACACAGGTGCCATTGTTGAA-3'	408 bp
Notch2	For: 5'- ACCCTTGTATGCACGGAGTC -3'<br>Rev: 5'- CCAGGTTATTGCACGTTCCT -3'	373 bp
Notch3	For: 5'-GCACCTGCAACCCTGTTTAT-3'<br>Rev: 5'-TCTCCAGCATCACCACAGAG-3'	370 bp
Ptcra	For: 5'- TCACACTGCTGGTAGATGGA-3'<br>Rev: 5'-TAGGCTCAGCCACAGTACCT-3'	364 bp
Rag1	For: 5'- ACCATGTGTCAAGCCACAAA-3'<br>Rev: 5'-TGGCTACAGCTGAGGAAGGT-3'	332 bp
Rag2	For: 5'-TCTCTAAAGATTCCTGCTACCTC-3'<br>Rev: 5'-TGGAATTCACTGCTGGGGTAC-3'	563 bp
Cd3e	For: 5'- CCTGTTCCCAACCCAGACTA-3'<br>Rev: 5'- AGGGCCAATTAGGAGAGGAA-3'	412 bp
Cd3z	For: 5'- ATCCCAGGGAAGCAGAAGAT -3'<br>Rev: 5'- TGTGCCGATCTCACTGTAGG -3'	404 bp
Gata3	For: 5'-TGAAGAAAGAAGGCATCCAG-3'<br>Rev: 5'-AACTCTTCGCACACTTGGAG-3'	320 bp
Id1	For: 5'- CCAGTGGGTCTCATCCCTTA -3'<br>Rev: 5'- AGAAATCCGAGAAGCACGAA -3'	368 bp
Id2	For: 5'- ACTCGCATCCCACTATCGTC -3'<br>Rev: 5'- TCCCCATGGTGGGAATAGTA -3'	453 bp

Runx1	For: 5'- GAGGCAAACTCTGTCCTGAA -3'<br>Rev: 5'- TTAGGCCTCAAAGACACCTG -3'	312 bp
Bcl11b	For: 5'- CCCCCAGCCTACAGATAAAT -3'<br>Rev: 5'- CGGGTCAACAGAATTCAAAC -3'	311 bp
Ebf1	For: 5'- TGCGGAAATCCAACTTCTTC -3'<br>Rev: 5'- GGTTCTTGTCTTGGCCTTCA -3'	257 bp
Pax5	For: 5'- GGGCTCCTCATACTCCATCA -3'<br>Rev: 5'- CGTCAAGTTGGCTTTCATGT -3'	321 bp
Pou2f2	For: 5'- GGAGCTGGAACAGTTTGCTC -3'<br>Rev: 5'- GATGCTGGTCCTCTTCTTGC -3'	310 bp
Sfp1	For: 5'- CGGATGACTTGGTTACTTACG -3'<br>Rev: 5'- GTAGGAAACCTGGTGACTGAG -3'	292 bp
Csf1r	For: 5'- ATGAGTCCCTCTTCACTCCG -3'<br>Rev: 5'- ACCTTCAGCACTGCATCTTC -3'	306 bp
Cebpa	For: 5'- CGCTGGTGATCAAACAAGAG -3'<br>Rev: 5'- TCACTGGTCAACTCCAGCAC -3'	499 bp
E12	For: 5'- TGACAGCTACAGCAGGGATG -3'<br>Rev: 5'- GAGTAGATCGAGGCCAGTGC -3'	444 bp
Cbfb	For: 5'- GAAGCTGATGCTGACCTTGT -3'<br>Rev: 5'- ACGCCAGCATTAAGACAGAC -3'	302 bp
Myb	For: 5'- CTTCCAGCTTCAGCAAAGAG -3'<br>Rev: 5'- GGAGGGTAAGGTAGGTGCAT -3'	331 bp
Ets1	For: 5'- TTCTCAGAAGCCTGTTGGAC -3'<br>Rev: 5'- AAACAGTTTTGGACCCCTTC -3'	290 bp
Ets2	For: 5'- CTCAACACCGTCAATGTCAA -3'<br>Rev: 5'- CTGGCTACAGTCCTCCTCAA -3'	232 bp
Adcy3	For: 5'- AGATGGAAACACGCTACTCG -3'<br>Rev: 5'- AACATTGGCCATAACCAGAA -3'	309 bp
Pde3b	For: 5'- CAGTAGCTTGATGGGTGCTT -3'<br>Rev: 5'- AGACGATGACCTCTGCTTTG -3'	341 bp
Creb	For: 5'- TGCCACATTAGCCCAGGTAT -3'<br>Rev: 5'- GTACCCCATCCGTACCATTG -3'	506 bp
Foxp3	For: 5'- TCTCCAGGTTGCTCAAAGTC -3'<br>Rev: 5'- CCAGGGGATAGTTCCTTGTT -3'	370 bp
Itga6	For: 5'- TGAGGTGTGTGAACATCAGG -3'<br>Rev: 5'- TAGAGCCAGCATCAGAATCC -3'	306 bp
Itgav	For: 5'- GGAGAACCAGAACCATTCCT -3'<br>Rev: 5'- TTGCTCTTCTTGAGGTGGTC -3'	272 bp
Itgb1	For: 5'- AAGACATGGACGCTTACTGC -3'<br>Rev: 5'- ATGGACCAGTGTCCAAAGAA -3'	267 bp
Itgb2	For: 5'- TAATGCAAGTTGCTGCATGT -3'<br>Rev: 5'- GCTGGAGTCGTCAGACAGTT -3'	332 bp
Itgb3	For: 5'- ATACCAGGGAGGACCTTCAG -3'<br>Rev: 5'- TCCTTCCCTGCTAGTTTCCT -3'	257 bp
Itgb4	For: 5'- GTCTGACGATCTGGACAACC -3'<br>Rev: 5'- CGTTCTCCTTGCAGTTTGTT -3'	246 bp
Pecam1	For: 5'- TTGGCACAACAAACAAGCTA -3'<br>Rev: 5'- GAAATCTTCTCGCTGTTGGA -3'	292 bp
Itga4	For: 5'- AAGCCAGCGTTCATATTCAG -3'<br>Rev: 5'- ATCCAGCCTTCCACATAACA -3'	277 bp

Itga5	For: 5'- GTACCTGGGTGACAAGAACG -3'<br>Rev: 5'- GTTCAGGTTCTTGCTGAGGA -3'	323 bp
Icam1	For: 5'- CCAAGAAACGCTGACTTCAT -3'<br>Rev: 5'- CGACCCTTATGAGAAAAGCA -3'	327 bp
Icam2	For: 5'- GAAGCCACAGAGTCTTGGAA -3'<br>Rev: 5'- TCAGTGTGACTTGAGCTGGA -3'	244 bp
Vcam1	For: 5'- CTGTACATCCCTCCACAAGG -3'<br>Rev: 5'- ACACGTCAGAACAACCGAAT -3'	321 bp
Itgae	For: 5'- AATGGCATTCAGTGGTCTGT -3'<br>Rev: 5'- TCCTTGTGCTCTCCAAGTTC -3'	346 bp
Sell	For: 5'- CGCTCATTCATCCCATTAAC -3'<br>Rev: 5'- GCAAGGAGTCTGAGTTTCCA -3'	224 bp
